# Postmenopause as a key factor in the composition of the Endometrial Cancer Microbiome (ECbiome)

**DOI:** 10.1038/s41598-019-55720-8

**Published:** 2019-12-16

**Authors:** Dana M. Walsh, Alexis N. Hokenstad, Jun Chen, Jaeyun Sung, Gregory D. Jenkins, Nicholas Chia, Heidi Nelson, Andrea Mariani, Marina R. S. Walther-Antonio

**Affiliations:** 1 Microbiome Program, Center for Individualized Medicine, Mayo Clinic, Rochester, Minnesota USA; 2 Division of Surgical Research, Department of Surgery, Mayo Clinic, Rochester, Minnesota USA; 3 Department of Obstetrics & Gynecology, Mayo Clinic, Rochester, Minnesota USA; 4 Department of Health Sciences Research, Mayo Clinic, Rochester, Minnesota USA; 5 Division of Rheumatology, Department of Internal Medicine, Mayo Clinic, Rochester, Minnesota USA; 6 Department of Molecular Pharmacology & Experimental Therapeutics, Mayo Clinic, Rochester, MN USA; 7 Division of Gynecologic Surgery, Mayo Clinic, Rochester, MN USA

**Keywords:** Microbiome, Endometrial cancer

## Abstract

Incidence rates for endometrial cancer (EC) are rising, particularly in postmenopausal and obese women. Previously, we showed that the uterine and vaginal microbiome distinguishes patients with EC from those without. Here, we sought to examine the impact of patient factors (such as menopause status, body mass index, and vaginal pH) in the microbiome in the absence of EC and how these might contribute to the microbiome signature in EC. We find that each factor independently alters the microbiome and identified postmenopausal status as the main driver of a polymicrobial network associated with EC (ECbiome). We identified *Porphyromas somerae* presence as the most predictive microbial marker of EC and we confirm this using targeted qPCR, which could be of use in detecting EC in high-risk, asymptomatic women. Given the established pathogenic behavior of *P. somerae* and accompanying network in tissue infections and ulcers, future investigation into their role in EC is warranted.

## Introduction

Endometrial cancer (EC) is the most common gynecological malignancy in the United States and the fourth most common cancer among women^1,2^. In addition, EC incidence rates are on the rise in the western world, suggesting that alterations in environmental factors such as diet, lifestyle, and the microbiome may be important drivers in EC etiology^3,4^. The increasing prevalence of obesity, and metabolic syndrome, along with the population aging are thought to contribute to this pattern^3^. Postmenopausal status and obesity are known risk factors for type I EC, which constitutes 70–80% of endometrial cancer cases (endometrioid morphology) that are often preceded by endometrial hyperplasia^5,6^. It is thought that postmenopausal women with a high body max index (BMI) are at an increased risk for Type I EC because of higher plasma estrogen^6^. In contrast, type II tumors are not sensitive to estrogen, generally contain mutations in the *p53* gene, and tend to be aggressive with a poor prognosis^5^. Type II EC develops in a background of endometrial atrophy, but hormonal risk factors are unknown, making early identification and treatment important to improved patient prognosis^5,7^. Importantly, Type II EC is less likely to manifest early through postmenopausal bleeding, and is more common among Black women^8^, contributing to the existing health disparities in the disease outcome^9^.

While prevailing hypotheses for EC etiology focus on the roles of obesity and estrogen, they do not consider the potential role of reproductive tract (RT) microbiota. Previous studies have demonstrated that microorganisms play important roles in cancer causation and development, ranging from cervical cancer^10^ to gastrointestinal malignancies^11^. For instance, in cervical cancer, changes in the vaginal microbiome aid in persistence of the human papillomavirus, a known cause of the disease^12^, and *Helicobacter pylori* is a known causative agent of gastric cancer^13^. The established role of the vaginal microbiome as a key factor in vaginal and obstetric health^12,14–20^, as well as vaginal microbiome differences found between different ethnicities^21^ adds to the importance of exploring the microbiome role in EC. We have previously found microbiome differences between patients with and without EC, namely an increased representation of *Porphyromonas somerae* and *Atopobium vaginae* in patients with EC^22^. Our goal in the present study is to understand how EC risk factors alter the RT microbiome and EC risk. Here we provide an in-depth examination of the influence of EC risk factors on the microbiome, correlate those with microbes enriched in EC, and evaluate the translational potential of these findings.

## Results

To find reproductive tract microbiome-based distinctions between patients with and without EC, we recruited women undergoing a hysterectomy for either endometrial cancer or a benign uterine condition at Mayo Clinic in Rochester, MN (IRB 12-004445). To understand the impact of patient risk factors (postmenopause and obesity) on microbiome composition, we first studied the microbiome of patients without EC, who had those risk factors present or absent. We then adjusted our analysis for these risk factors in order to identify the EC polymicrobial network (ECbiome). We further took those findings and developed a qPCR test to discriminate patients with EC from those without based solely on the microbiome results. In addition, to determine if ECbiome microorganisms are likely to be a factor early in the disease progress (complex hyperplasia with atypia), or aggressive manifestations of the disease (Type II EC), we investigated the microbiome signatures of these subgroups.

### Patient demographics

Microbiome samples (see Methods for details) were collected from 165 patients with a variety of uterine conditions. Hysterectomy tissue samples were collected from half of these patients. Of the total patients, 148 had sufficient quantity and quality of DNA to produce successful sequencing results. There were 75 patients without EC, 66 were diagnosed with EC (56 with Type I endometrioid, and 10 with Type II non-endometrioid), and 7 with complex atypical hyperplasia (Table 1). The patient cohort recapitulated known EC risk factors, with EC patients being significantly older (*p* < 0.001), predominantly postmenopausal (*p* < 0.001), and with a higher body mass index (BMI, *p* = 0.004). In addition, EC patients were significantly more likely to have a high vaginal pH (>4.5, *p* < 0.001), which is consistent with our previous report^22^. A history of diabetes was not significantly associated with patients with EC (*p* = 0.092).

**Table 1:** Patient clinical characteristics. Data is presented as mean (standard deviation).

	Benign	EC	*P* value	Hyperplasia
	(N = 75)	(N = 66)	(Benign vs Cancer)	(N = 7)
Age (in years)	49.9 (10.5)	61.8 (10.3)	2.82E-10	55.0 (3.3)
BMI (kg/m^2^)	30.8 (8.4)	35.2 (9.3)	0.004	39.9 (11.8)
**Ethnicity (%)**				
Caucasian	96	97	NA	85.7
African American	0	0		14.3
Native American	1.3	3		0
Filipino	1.3	0		0
Chinese	1.3	0		0
**Menopausal Status (%)**				
Pre/Peri	73.3	16.7	5.39E-11	42.9
Post	26.7	83.3		57.1
**History of Diabetes (%)**				
Yes	10.7	22.7	0.092	0
No	85.3	74.2		100
Unknown	4	3.1	NA	0
**Obese** + **Diabetic (%)**				
Yes	6.7	21.2	0.028	0
Not diabetic	85.3	74.2		100
Unknown	8	4.6		0
Vaginal pH (%)*				
Normal (≤4.5)	37.5	1.8	5.67E-06	57.1
High (>4.5)	62.5	98.2		42.9
**Indication for Hysterectomy (%)**				
Endometrial cancer	0	89.3	NA	0
Hyperplasia with/without atypia	0	6.1		85.7
Benign - No abnormal bleeding	24	0		14.3
Benign - Abnormal bleeding	58.7	0		0
Unknown	17.3	4.6		0
**FIGO Grade (%)**				
G1/G2	0	72.7	NA	0
G3	0	21.2		0
Uknown	0	6.1		0
**Histotype (%)**				
Adenocarcinoma endometrioid	NA	84.8	NA	NA
Adenocarcinoma serous	NA	7.6		NA
Adenocarcinoma clear cell	NA	1.5		NA
Adenocarcinoma squamous	NA	0		NA
Carcinosarcoma	NA	6.1		NA
**FIGO Stage 2009 (%)**				
IA	NA	72.7	NA	NA
IB	NA	15.2		NA
II	NA	0		NA
IIIA	NA	0		NA
IIIC1	NA	4.5		NA
IIIC2	NA	1.5		NA
IV	NA	3		NA
Unknown	NA	3.1		NA

Statistical significance assessed by *t* test for continuous covariates and chi-squared test for categorical covariates. *Twenty-two patients not included in this section as they have unknown vaginal pH values. EC = Endometrial cancer. BMI = body mass index FIGO = International Federation of Gynecology and Obstetrics.

### Impact of risk factors on microbiome composition in patients without EC

#### Postmenopausal patients exhibit significantly increased microbiota diversity with enrichment of anaerococcus, peptoniphilus, and porphyromonas species

**Figure 1:**
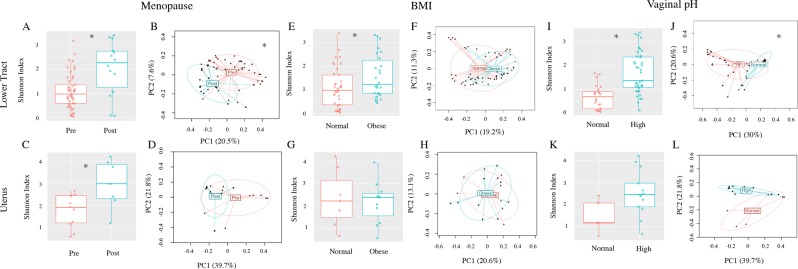
Influence of patient factors on bacterial community diversity among patients without EC. Both α- (Shannon index) and β-diversity measures were compared. For α-diversity a Wald statistical test was performed. For β-diversity, omnibus *p* values are reported combining the evidence across the Bray-Curtis, unweighted, weighted, and generalized UniFrac distance metrics. The most significant metric is shown in each ordination plot. Lower tract (cervix and vagina). (**A**) Pre vs post-menopause α-diversity *p* = 0.002. (**B**) Pre vs post-menopause β-diversity unweighted UniFrac (omnibus *p* = 0.007), uterus. (**C**) Pre vs post-menopause α-diversity *p* = 0.024. (**D**) Pre vs post-menopause β-diversity weighted UniFrac (omnibus *p* = 0.221), lower tract. (**E**) Obese vs normal BMI α-diversity *p* = 0.019. (**F**) Obese vs normal BMI β-diversity unweighted UniFrac (omnibus *p* = 0.09), uterus. (**G**) Obese vs normal BMI α-diversity *p* = 0.47. (**H**) Obese vs normal BMI β-diversity unweighted UniFrac (omnibus *p* = 0.514), lower tract. (**I**) Normal vs high vaginal pH α-diversity *p* = 2.871E^−5^. (**J**) Normal vs high vaginal pH β-diversity generalized UniFrac (omnibus *p* = 0.001) and uterus. (**K**) Normal vs high vaginal pH α-diversity *p* = 0.112. (**L**) Normal vs high vaginal pH β-diversity weighted UniFrac (omnibus *p* = 0.062). Uterus premenopause N = 11, postmenopause N = 7; Lower tract premenopause N = 49, postmenopause N = 14. Uterus normal BMI N = 7, obese BMI N = 11; Lower tract normal BMI N = 38, obese BMI N = 26. Uterus normal pH N = 5 and high pH N = 13; Lower tract normal pH N = 24, high pH N = 39. For each primary comparison, the PERMANOVA tests were adjusted for the two remaining factors (menopause status, pH, obesity). *Groups are significantly different.

Based on the significant differences we found among patient factors, we examined their impact on the α- (within samples) and β- (between samples) diversity in the microbial community composition among patients without EC (Fig. 1).

**Figure 2:**
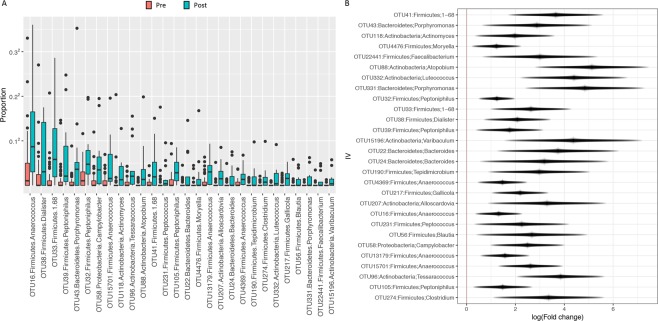
Specific bacterial OTUs differentially enriched among the lower tract of patients without EC by menopause status. (**A**) Proportion of OTUs significantly enriched among post-menopause patients. (**B**) Effect plot of the enriched OTUs. Differential abundance analysis was adjusted for vaginal pH and obesity. Wald statistical test with Q value cutoff = 0.1.

We focused on the Shannon diversity index (i.e., community richness and evenness) as the representative measure for α-diversity and used an omnibus test (see Methods *α & β-Diversity*) to combine the evidence of four β-diversity metrics. In the lower tract, both α- and β-diversity were associated with patient menopause status (*p* = 0.002 and *p* = 0.007, respectively; Fig. 1A,B), with postmenopausal patients exhibiting increased microbiota diversity. In the uterus, the microbiome α-diversity (*p* = 0.024; Fig. 1C), reached significance while β-diversity did not (*p* = 0.221; Fig. 1D). However, based on the similarity in trends between the lower tract and uterus, the lack of significance within the uterus β-diversity is likely due to lower sample numbers from this organ. Additional β-diversity measures are shown in Supplemental Fig. S1. We then performed differential abundance analysis (adjusted for all other significant confounders, see Methods *Differential Abundance Analysis* for details) to identify which taxa drove the verified microbiome structural changes in the vaginal microbiome of postmenopausal women. There were 28 taxa found significantly enriched in postmenopausal women (Q < 0.1) including *Anaerococcus, Peptoniphilus*, and *Porphyromonas* species (Fig. 2 and Supplemental Table S1). We attempted to inquire about estrogen therapy use in our patients through a questionnaire about current and prior estrogen use and electronic medical record (EMR) review in order to discern any significant impact in the microbiome composition. Unfortunately, nearly all postmenopausal patients (85%) did not respond to this question and it was absent from their medical record (which could indicate no use or missing data). Because this information could not be verified in the EMR and due to the low number of patients with unambiguous data we refrained from pursuing this information as statistically meaningful.

#### BMI increases microbiota diversity in the lower tract

BMI associations with the microbiome (normal vs obese – BMI ≥30) were significant for α-diversity in the lower tract (*p* = 0.019, Fig. 1E), with obese women displaying increased microbiome diversity. However, the β-diversity did not reach significance (*p* = 0.09, Fig. 1F). For uterus microbiome, BMI was not a significant source of variation (uterus α-diversity, *p* = 0.47, Fig. 1G; uterus β-diversity, *p* = 0.514 Fig. 1H). Among the additional β-diversity tests (Supplemental Fig. S1), unweighted UniFrac analysis of BMI was significant in the lower tract (*p* = 0.035). This indicates that BMI has more of an impact on rarer taxa, since the unweighted UniFrac places more emphasis on rare and less abundant taxa^23^.

#### High vaginal pH significantly increases lower tract microbiota diversity and alters community structure

Vaginal pH (high, >4.5 vs normal, ≤4.5) was associated with significant microbiome changes in the lower tract microbiome (α-diversity *p* < 0.001, Fig. 1I and β-diversity *p* = 0.001, Fig. 1J), with high vaginal pH significantly associated with increased microbiota diversity. The α-diversity and β-diversity did not reach significance in the uterus (*p* = 0.112, Fig. 1K; *p* = 0.062, Fig. 1L). Taxa differential abundance analysis of the vaginal microbiome (adjusted for all confounders) did not indicate any significantly enriched OTUs at the level of Q < 0.1.

In summary, all parameters analyzed (menopause status, BMI, and vaginal pH) showed a significant impact on the microbiome composition, with postmenopause, obesity, and high vaginal pH significantly increasing the microbiome diversity. Of note is the marked enrichment of *Anaerococcus, Peptoniphilus*, and *Porphyromonas* species by postmenopausal status.

### Microbiome in patients with endometrial cancer (ecbiome)

#### The ecbiome

**Figure 3:**
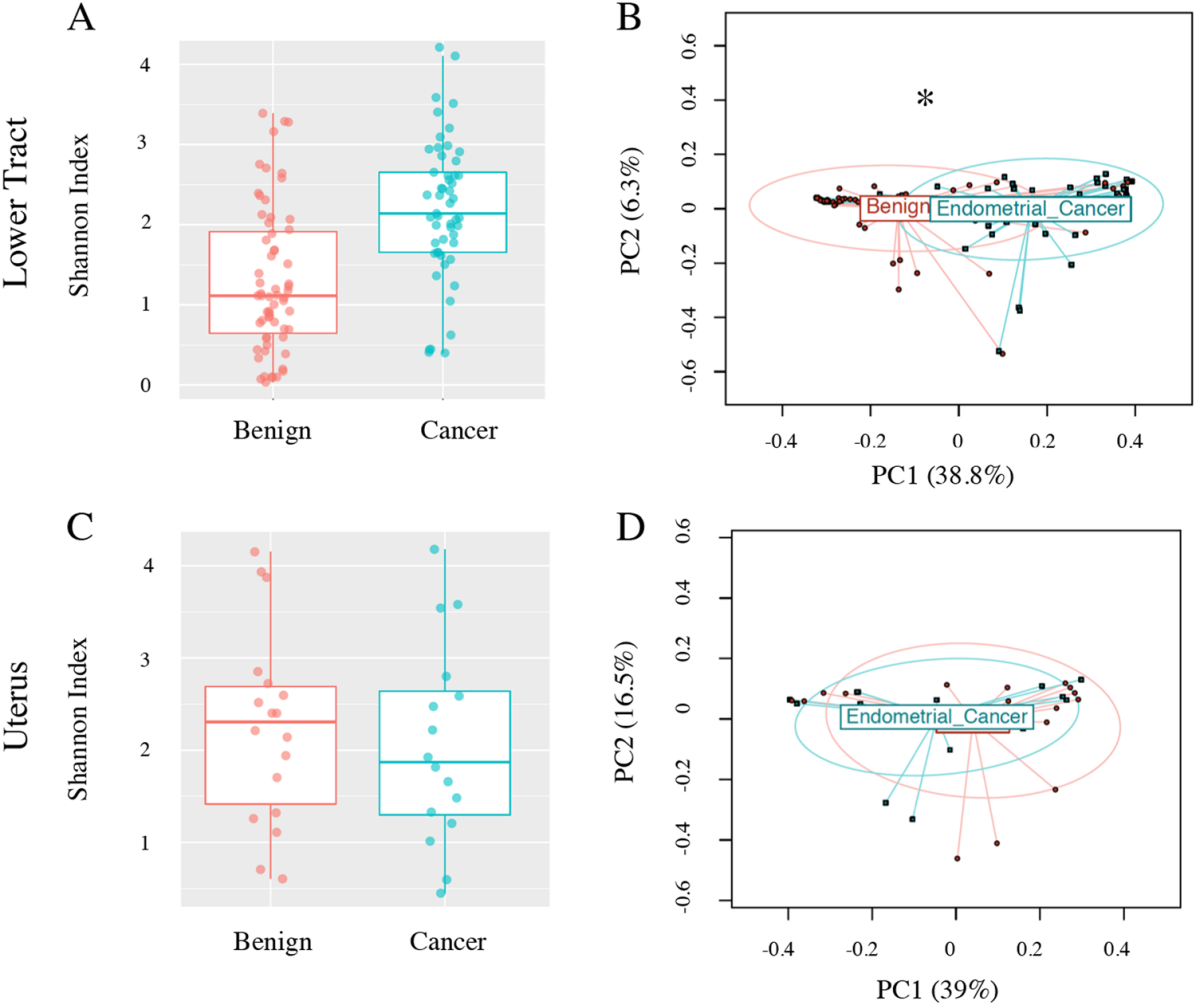
Bacterial community α- and β-diversity among patients with and without EC. Both α- (Shannon index) and β-diversity measures were compared. For α-diversity a Wald statistical test was performed. For β-diversity, omnibus *p* values are reported combining the evidence across the Bray-Curtis, unweighted, weighted, and generalized UniFrac distance metrics. The most significant metric is shown in each ordination plot. (**A**) Lower tract benign vs cancer, α-diversity *p* = 0.417, (**B**) lower tract benign vs cancer, β-diversity weighted UniFrac (omnibus *p* = 0.04), (**C**) Uterus benign vs cancer, α-diversity *p* = 0.168, (**D**) uterus benign vs cancer, β-diversity weighted UniFrac (omnibus *p* = 0.194). Uterus cancer = 16, benign = 18, lower tract cancer N = 54, benign N = 64. ANOVA and PERMANOVA tests were adjusted for menopause status, vaginal pH, and obesity. *p ≤ 0.05.

After adjustment for all significant parameters (see Methods), patients with endometrial cancer were not found to have a significantly different α-diversity than patients without endometrial cancer (lower tract *p* = 0.417, Fig. 3A; uterus *p* = 0.168, Fig. 3C).

**Figure 4:**
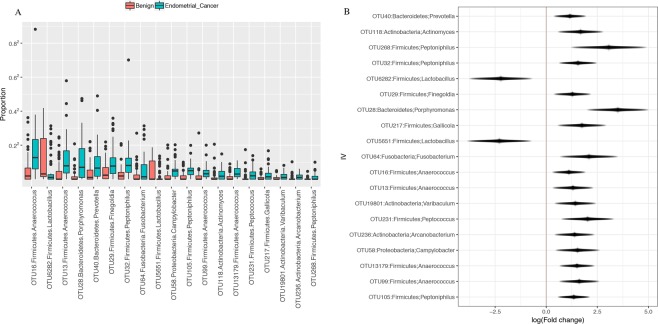
Significantly enriched taxa among patients with and without cancer. (**A**) Proportion of significantly enriched OTUs, (**B**) Effect size plot of enriched OTUs. Analysis was adjusted for pH, menopause status, and BMI. Benign N = 67, Cancer N = 57. Samples rarefied prior to analysis. Wald statistical test with Q value cutoff = 0.1.

**Figure 5:**
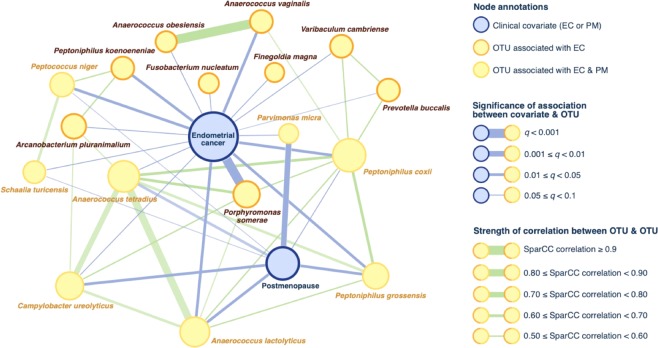
Network depiction of significant associations between endometrial cancer and ECbiome (17 taxa associated with endometrial cancer). In this network, nodes represent OTUs and clinical covariates; edges link either (i) an OTU with its statistically-associated clinical factor, or (ii) correlated OTUs found using SparCC on the entire OTU compositional (i.e. relative abundances) table. Eight of the 17 ECbiome taxa are independently enriched by postmenopause, which is depicted in this figure to clarify relationships. The microbe most strongly associated with endometrial cancer (*P. somerae*) is not associated with postmenopause. Species-level annotation for each OTU was determined by identifying its phylogenetically closest bacterial genome using BLAST.

However, significance was found in β-diversity of the lower tract (*p* = 0.04, Fig. 3B). No significance was found in the β-diversity of the uterus (*p* = 0.194, Fig. 3D), although weighted Unifrac distance trended towards significance (*p* = 0.07, Supplemental Fig. S2**)**, indicating more relevance of the dominant taxa in that trend. Differential abundance analysis of the lower tract showed that there were 17 taxa found significantly enriched in patients with EC (ECbiome, Fig. 4, Supplemental Tables S2 and S3). Of note, 8 of the OTUs found enriched in patients with EC were also found enriched in postmenopausal patients without EC. The remaining 9 OTUs were found to be solely enriched by EC. Two *Lactobacillus iners* OTUs were found depleted in patients with EC (OTUs 5651 and 6282). To summarize these significant relationships in a systems-level context, we constructed a network model portraying the links within and between the ECbiome and postmenopause (Fig. 5).

The network displays a complex net of relationships, and identifies *P. somerae* as the microbe with the strongest association with EC. Of notice, *P. somerae* is not associated with postmenopause. However, the correlation network of this microbe identified connections to 4 other microorganism (*A. tetradius, A. lactolyticus, P. coxii*, and *C. ureolyticus*), which are associated with postmenopause, and represent the most connected nodes in the network.

#### Porphyromonas somerae as an indicator of EC

**Figure 6:**
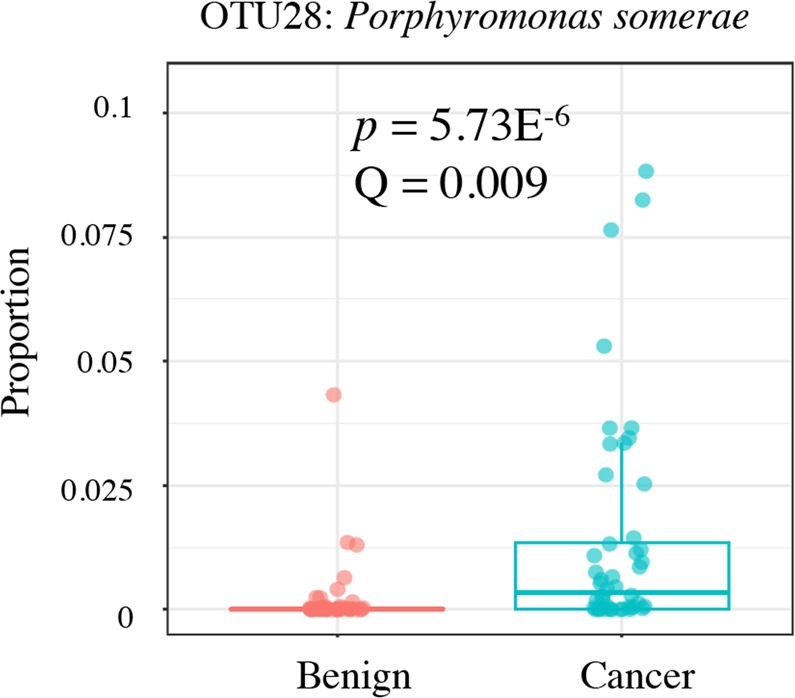
Significant enrichment of *Porphyromonas somerae* in endometrial cancer. Proportion of significantly enriched OTU28 (*P. somerae*) among cancer and benign patients. Benign N = 67, Cancer N = 57. Samples rarefied prior to analysis. Wald statistical test.

**Figure 7:**
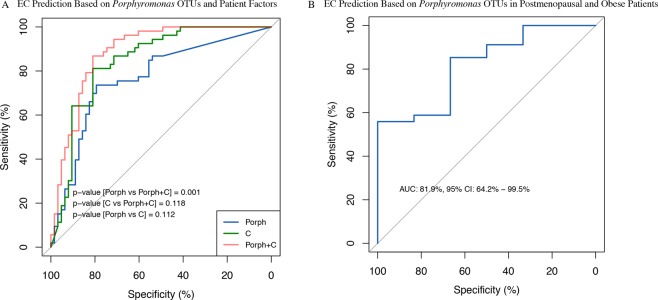
Predictive potential of *Porphyromonas* OTUs in endometrial cancer. (**A**) Receiver operator characteristic (ROC) curve for the ability of all *Porphyromonas* OTUs (Porph; AUC = 76.7%, 95% CI 67.9–85.5%), patient factors alone. (**C**) AUC = 84.4%, 95% CI 77.1–91.7%), and *Porphyromonas* OTUs and patient factors together (Porph + C; AUC = 88.6%, 95% CI 82.4–94.7%) to predict EC. Patient characteristics included in the ROC curve were patient age, BMI, menopause status, and vaginal pH. Benign N = 67, EC N = 57. (**B**) ROC curve for the ability of all *Porphyromonas* OTUs to predict EC in the high-risk post-menopausal and obese patients (AUC = 81.9%, 95% CI 64.2–99.5%). Benign N = 5, EC N = 35. Samples rarefied prior to analysis. CI = confidence intervals.

*P. somerae* emerges as the most significantly enriched species in patients with EC (*p* < 0.001, Q = 0.009, Fig. 6). To further inquire about the significance of this finding, we created a receiver operator characteristic (ROC) curve to determine whether detection of *Porphyromonas* OTUs by sequencing could predict EC status (Fig. 7A).

We included all *Porphyromonas* OTUs rather than only *P. somerae* to avoid overfitting. Prediction of EC by *Porphyromonas* OTUs alone gives an area under the curve (AUC) of 76.7% (95% confidence intervals (CI): 67.9–85.5%). The patient factors (menopause status, obesity, and vaginal pH) alone give an AUC of 84.4% (95% CI: 77.1–91.7%). When combined together, *Porphyromonas* detection and the risk factors give an AUC of 88.6% (95% CI: 82.4–94.7%).

#### The microbiome of the lower reproductive tract and uterus are significantly different but correlated in patients with ec

The microbiome of the lower tract and uterus was significantly different for patients with (*p* = 0.027, Supplemental Table S4) and without (*p* = 0.02, Supplemental Table S5) EC. However, the microbiome of the lower tract was correlated with the uterine microbiome in patients with EC (*p* = 0.01, Supplemental Table S6), indicating that the vaginal microbiome can be used as a proxy of the uterine microbiome in these patients. This correlation was not identified in patients without EC (*p* = 0.111, Supplemental Table S7). The comparative microbiome profile for all sample sites is shown in Supplemental Figs. S3 **(**patients without EC) and S4 (patients with EC), displaying general dominance of lactobacilli species except for within stool, where *Bacteroidetes* dominate. Of note is the prominence of *Prevotella* species in the uterus of women without EC, which is not verified in the Fallopian tubes or ovaries.

#### Confirmation of *P. somerae* correlation with EC by qPCR of vaginal samples

**Figure 8:**
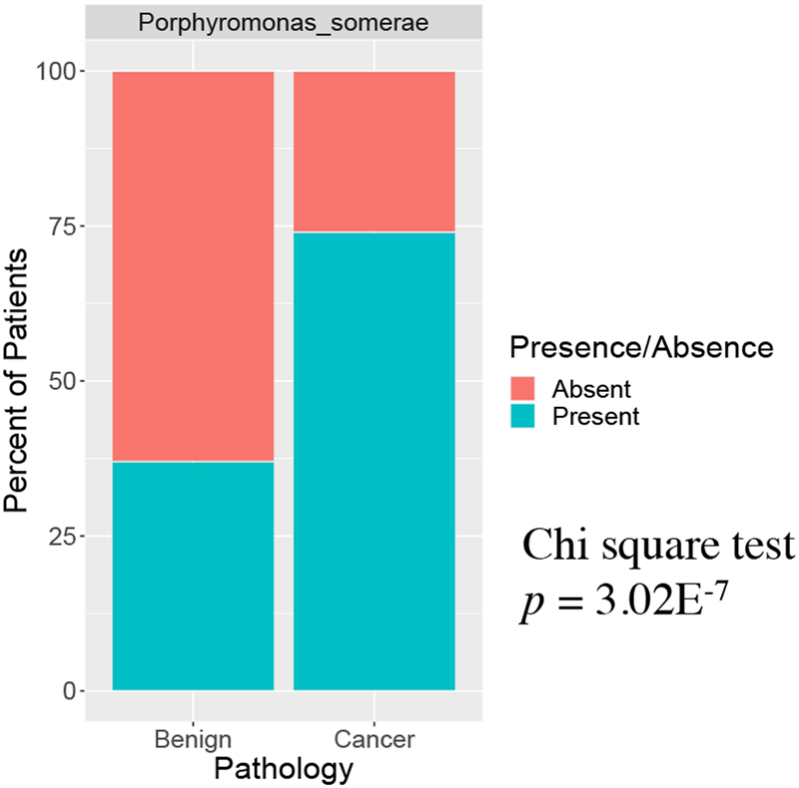
Significant detection of *Porphyromonas somerae* in endometrial cancer by qPCR. qPCR detection of *P. somerae* among vaginal swabs of patients with and without cancer. Benign N = 73, Endometrial Cancer N = 65. Samples rarefied prior to analysis.

To confirm our BLAST and sequencing results, we developed a qPCR assay to specifically detect this bacterial species in the vagina of EC patients. Our previous work showed that approximately 1 μg of microbial DNA could be isolated from each vaginal swab^22^. qPCR experiments revealed a lower limit of detection of 1 × 10^−5^ ng/μL for *P. somerae* (Supplemental Fig. S5). As a control we used *A. vaginae* with a limit of detection of 1 × 10^−6^ ng/μL in pure culture (Supplemental Fig. S6**)** because we had found a statistical association between this microbe and EC in our prior cohort^22^. *A. vaginae* was not found to be associated with EC in this cohort (Supplemental Table S8), although *Atopobium* species were associated with postmenopausal status **(**Supplemental Table S1**)**. Vaginal swabs from the initial cohort of 31 patients whose sequencing data were published in our pilot study^22^ were used as a confirmatory test. After validation of probe specificity, we amplified bacterial DNA from patient vaginal swabs. An overall total of 146 patients were tested using the qPCR assay. Of these, *P. somerae* was present in 74% of the 65 EC patients and 37% of the 73 patients without EC (Fig. 8 and Supplemental Table S9; *p* = 3.02E^−7^). Among all samples with positive qPCR results for *P. somerae*, the frequency of distribution between patients with and without cancer was significantly different, with more EC patients showing *P. somerae* detection in both sequencing and qPCR results (Supplemental Fig. S7). Among all patients, the sensitivity of *P. somerae* as a predictive marker of EC was 74%, with a specificity of 63% (Supplemental Table S10). When we focused on patients at high risk of EC (obese and postmenopausal) the positive predictive value increased to 0.86.

#### P. somerae present in all type II EC cases

To evaluate whether our findings may extend to Type II EC (non-endometrioid, and most aggressive manifestation of EC), and precancerous transformations (endometrial hyperplasia), we investigated the distribution of the 17 EC enriched OTUs in these subgroups (9 patients with Type II EC and 7 patients with complex hyperplasia with atypia). The results in Table 2 show that all of the 17 OTUs are enriched in Type II EC when compared to the patients without EC, in alignment with Type I EC. That is also the case for patients with complex hyperplasia with atypia, although less noticeable. This data supports a microbiome progression towards the enrichment of these 17 OTUS, from the absence of EC, to a precancerous manifestation, and into EC, regardless of the Type. Of particular notice is that *P. somerae* was detected in all (100%) Type II EC patients, and 57% of the hyperplasia patients Supplemental Table S11. This indicates that the association of this microorganism with EC is inclusive of Type II and may be even stronger for non-endometrioid types.

**Table 2:** Median abundance of EC-enriched OTUs in patients with benign pathology, hyperplasia, and Types I and II EC.

OTU ID	Blast ID	Median			
		Benign	Hyperplasia	Type I EC	Type II EC
40	*Prevotella buccalis*	0.023% [0.004%,0.13%]	0.212% [0.001%,0.916%]	0.533% [0.263%,1.169%]	0.374% [0.007%,5.222%]
28	*Porphyromonas somerae*	0.001% [0%,0.005%]	0.005% [0%,0.261%]	0.43% [0.045%,1.251%]	0.665% [0.049%,10.993%]
64	*Fusobacterium nucleatum*	0.001% [0%,0.003%]	0.03% [0.001%,0.229%]	0.05% [0%,0.177%]	0.004% [0%,0.378%]
58	*Campylobacter ureolyticus*	0.002% [0%,0.006%]	0.029% [0%,0.333%]	0.234% [0.063%,0.301%]	0.223% [0.112%,0.263%]
231	*Peptococcus niger*	0% [0%,0%]	0.001% [0%,0.016%]	0.043% [0.002%,0.118%]	0.066% [0%, 0.142%]
13	*Anaerococcus obesiensis*	0.013% [0.003%,0.095%]	0.053% [0%,0.171%]	0.637% [0.258%,1.631%]	0.559% [0.145%,4.198%]
99	*Anaerococcus vaginalis*	0.002% [0.001%,0.009%]	0.014% [0%,0.048%]	0.095% [0.056%,0.193%]	0.102% [0.022%,0.361%]
16	*Anaerococcus tetradius*	0.031% [0.005%,0.12%]	0.366% [0.002%,1.08%]	1.613% [0.805%,3.597%]	1.212% [0.244%,6.883%]
13179	*Anaerococcus lactolyticus*	0.001% [0%,0.004%]	0.01% [0%,0.032%]	0.101% [0.057%,0.206%]	0.117% [0.022%,0.22%]
29	*Finegoldia magna*	0.054% [0.022%,0.137%]	0.185% [0.002%,0.455%]	0.609% [0.227%,1.204%]	0.527% [0.006%,1.747%]
217	*Parvimonas micra*	0% [0%,0.001%]	0.001% [0%,0.111%]	0.014% [0.001%,0.074%]	0.016% [0.001%,0.093%]
105	*Peptoniphilus coxii*	0.003% [0%,0.013%]	0.025% [0%,0.12%]	0.255% [0.1%,0.363%]	0.145% [0.039%,0.427%]
32	*Peptoniphilus grossensis*	0.039% [0.009%,0.068%]	0.24% [0.002%,0.337%]	0.483% [0.252%,0.816%]	1.211% [0.227%,4.849%]
268	*Peptoniphilus koenoeneniae*	0% [0%,0%]	0% [0%,0.002%]	0.004% [0.001%,0.02%]	0.002% [0%,0.02%]
19801	*Varibaculum cambriense*	0% [0%,0.002%]	0.005% [0.001%,0.044%]	0.016% [0.006%,0.04%]	0.01% [0.001%,0.02%]
118	*Schaalia turicensis*	0% [0%,0%]	0.001% [0%,0.026%]	0.014% [0.006%,0.107%]	0.068% [0%,0.588%]
236	*Arcanobacterium pluranimalium*	0% [0%,0%]	0.005% [0%,0.025%]	0.018% [0.002%,0.046%]	0.005% [0%,0.039%]

Darker red = higher abundance as compared to benign. These EC-enriched OTUs were identified based on the Wald test (Overdispersed Poisson regression, Benign vs. EC) with q < 0.1. 95% bootstrap percentile confidence intervals presented in square brackets.

## Discussion

The steadily increasing incidence rate of EC underlines the need for additional research into the disease to understand its etiology and develop better predictors of risk^24^. Our study confirms the association of previously known risk factors (postmenopausal status and obesity) with EC and identifies high vaginal pH (>4.5) as an additional risk factor. As we delved into the putative role of the microbiome in the risk profile for the disease, we found that all of the factors above (postmenopause status, obesity, and high vaginal pH) are significant modifiers of the reproductive tract microbiome composition, by increasing its diversity. While the paradigm in gut microbiome supports the notion of high microbiome diversity as an indicator of gut health, previous work, including our own, has established that a healthy vaginal microbiota is commonly low in microbial diversity and is dominated by *Lactobacilli* species^16^. Therefore, the increase in microbiome diversity we saw associated with postmenopause, obesity, and high vaginal pH can be interpreted as detrimental to reproductive tract health. Postmenopause seems to be a particularly impactful factor, specifically enriching for taxa commonly associated with unhealthy and symptomatic conditions in the reproductive tract.

More importantly, of the 17 taxa we found enriched in EC patients, 8 were also enriched by postmenopause. Because postmenopausal status is a main risk factor for endometrial cancer, this system can be thought of as an ecological succession towards a disease state. Though in-depth studies on the postmenopause RT microbiota have not been done, it is not surprising that menopause impacts the microbiome, given the complex interactions of estrogen, glycogen, and lactobacilli. High levels of estrogen stimulate glycogen-dependent metabolism within the vaginal mucosa, leading to increased colonization by lactobacilli^25^. Lactobacilli also metabolize glycogen to produce lactic acid, contributing to a low vaginal pH that is thought to reduce the risk of infection^16,26^. These bacteria also produce anti-microbial compounds and hydrogen peroxide, which act as antagonists to other bacteria^27,28^. This acidic environment they help to create in the vagina may act as a selective gatekeeper to the rest of the reproductive tract, preventing pathogenic organisms from colonizing and aiding in maintenance of organ-specific communities. In postmenopausal women, ovarian estrogen production has ceased, leading to lower glycogen levels, a propensity for lower lactobacilli colonization, and more basic vaginal pH levels, which may help explain the significant difference in pre- and postmenopausal RT microbiota. In a scenario where the postmenopausal transition is accompanied by obesity and a high vaginal pH, it is very likely that a shift in the reproductive tract microbiome towards increased diversity will occur, given that all of these factors independently increase the microbiome diversity, according to our results. Postmenopause seems to be a particularly defining factor, because it favors the enrichment of nearly half of the species associated with EC (ECbiome). It is possible that the species favored by postmenopause are not themselves involved in EC, but may be important first colonizers that facilitate the colonization by others that are more specifically associated with EC. Although our data does not allow us to infer the directionality of these relationships, it supports further investigation into the role of the identified ECbiome in the disease, which could be studied in a longitudinal setting.

Organisms within the ECbiome have been previously identified as polymicrobial associations in chronic soft tissue infections^29–31^, ulcers^32,33^, morbid obesity^34,35^ and bacterial vaginosis^36^. This suggests that this microbiome network is likely involved in mechanisms of host disease. Within the ECbiome, *Porphyromonas somerae* stood out because of its very significant and specific association with EC (OTU28, *p* = 5.73E-6; Q = 0.009). We had previously identified this species as significantly associated with EC^22^, and hereby validate that finding. However, in contrast with our prior cohort, we did not find *A. vaginae* prevalence to be statistically associated with EC, even though it was associated with postmenopausal status. As mentioned in our prior work^22^, *A. vaginae* is a well described vaginal pathogen, with known association with bacterial vaginosis^37^. Because this is a condition of difficult diagnosis, it is possible that some of the women with EC in our prior cohort had undiagnosed bacterial vaginosis, which may have led to that association. It is also possible that the ecological role played by *A. vaginae* can be substituted by other microbes in different individuals.

*P. somerae* has been isolated from diabetic foot ulcers, bacterial vaginosis, chronic otitis media, and chronic bone and soft tissue infections, suggesting that it may act as a pathogen^29,38^. Further, *P. gingivalis*, which is closely related to *P. somerae*, has been implicated in esophageal cancer^39^ and Alzheimer’s disease^40^, implying that the *Porphyromonas* genus contains species with the ability to cause serious disease in humans. The 4 ECbiome taxa we found correlated with *P. somerae* (*A. tetradius, A. lactolyticus, P. coxii*, and *C. ureolyticus*), are also the most connected nodes to other ECbiome taxa, indicating their putative prominent role in the disease processes. It is possible that they play a key role as first colonizers that facilitate or enable the subsequent colonization of *P. somerae* and others. Because the association of *P. somerae* with EC was robust and specific enough to potentially be considered as a biomarker for the disease (AUC 76.7%, CI: 67.9–85.5%), we developed a *P. somerae*-specific qPCR assay to detect this species in vaginal swabs. This becomes of particular interest because while we found that the lower tract and uterine microbiome are significantly different from each other in EC (*p* = 0.027), they are also correlated in these patients (*p* = 0.01), indicating that the vaginal microbiome can be used as a proxy for the uterine environment in EC. In agreement with the 16S rRNA gene sequencing data, *P. somerae* was detected significantly more frequently in vaginal swabs of patients with EC than those without EC (*p* = 3.02E-7). Overall, the test achieved a sensitivity of 74% and specificity of 63%, with a positive predictive value of 0.86 for obese and postmenopausal patients. These numbers approach those for the association of *Helicobacter pylori* with gastric cancer (sensitivity, 90%^41^; specificity, 50%^13^) and human papilloma virus with cervical cancer (sensitivity, 95%; specificity, 60%)^10^. *P. somerae* detection could therefore be pursued for development as a potential EC biomarker test for patients at high risk of developing EC, who may not experience or recognize early disease symptoms, which could help address marked health disparities that exist in EC^9^. This becomes of particular interest to pursue given that *P. somerae* was detected in all Type II EC cases in our study, which is more prevalent in black women^8,42^. Given that the vaginal microbiome of black women is often characterized by a higher diversity index^15^, the colonization by the ECbiome and *P. somerae* in particular may be facilitated, which could lead to a higher propensity for the development of microbiome derived cancer transformations.

### Limitations

Though we have examined the RT microbiota of women without EC, these were not healthy women. These hysterectomy patients came to Mayo Clinic due to abnormal bleeding, pain, or some combination of these. Though we can sample the vaginal tract of healthy women, we cannot perform hysterectomies or biopsies on healthy women. Further, we wanted to sample women undergoing the same procedures as EC patients. Regardless, we were able to identify differences in the microbiota between patients without and those with EC.

We describe the composition of the RT as a whole but we have few ovarian, fallopian tube, and uterine samples. While previous work clearly indicates lactobacilli dominance of the lower tract, it is less clear what bacteria are present further inside the reproductive tract^16^. A recent study suggests the presence of a more diverse microbiota within the ovaries and fallopian tubes, in agreement with our work, but at a much lower biomass than that of the low-diversity vagina^22,43^. Abundance of *Lactobacilli* among women without EC in our study was lower in the upper RT but still accounted for a majority of the identified microbial sequences. Although microbiome differences between the uteri of women with and without EC neared significance (weighted Unifrac *p* = 0.07), it did not reach a significant threshold. Given the small sample size of endometrial tissue samples (18 benign and 16 EC) and trend towards significance we believe that the lack of significance is due to undersampling. In the uterus of patients without cancer, the genus *Prevotella* was nearly equal to *Lactobacilli* abundance, but *Lactobacilli* dominate the fallopian tube and ovary microbiota, at least in the small number of samples we obtained. These differences in abundance of *Lactobacilli* between the uterus and lower tract suggests differences in levels of acidity between these locations. Given that these are not gynecologically healthy women, however, we cannot determine whether this represents a healthy RT microenvironment. Additional sampling of these areas would give added confidence to the results. Longitudinal sampling would also add to the study, as it would capture the spectrum of changes within premenopausal women and determine the stability of the postmenopausal RT microbiota. Studies will be needed to validate our microbial qPCR test in asymptomatic and healthy women. However, the fact that our control (non-EC) population is not a healthy population may be a strength in that the microbiome association is specific to EC and is distinct from various benign uterine diseases. Also, our study population was primarily white, so additional studies will be needed to confirm or modify the validity of findings in an ethnically representative population, in order to address existing health disparities in EC.

A caveat underlining the association of a high vaginal pH with EC is the possibility of microscopic amounts of blood present in the vagina that could falsely increase the pH value. We did not measure vaginal pH when blood was visible in the sample, but given that vaginal bleeding is a hallmark symptom of EC, we cannot rule out the possibility of the presence of microscopic amounts. The impact of this possibility is lessened by the fact that over half (54%) of patients without endometrial cancer also reported vaginal bleeding. It is not clear from this cross-sectional study whether EC leads to high vaginal pH or vice versa.

## Conclusions

Overall, we have verified the main known risk factors for EC (postmenopausal status and obesity) and identified high vaginal pH (>4.5) as an additional factor associated with patients with EC. We have determined that all of these factors impact the reproductive tract microbiome by increasing its diversity, moving it towards a loss of dominance by lactobacilli species. We further identify postmenopause as a key factor of taxa associated with EC (ECbiome). We highlight the signature presence of *Porphyromonas somerae* in patients with EC and discuss potential translational applications of this knowledge, which may bring new approaches to address current health disparities in EC.

## Methods

### Ethics statement

The Mayo Clinic Institutional Review Board (IRB) approved 2 protocols: #12-004445 (approved 8/13/2012) to perform sequencing and for patient enrollment with informed written consent being provided by all patients, and #15-000842 (approved 3/31/2015) for the use of patient samples to develop the qPCR assay. Patients were recruited from 01/05/2013 to 11/18/2015. All patients provided written informed consent for the utilization of their samples for future research studies within Mayo Clinic. All methods and procedures were performed in accordance with the Mayo Clinic IRB guidelines and regulations.

### Patient enrollment

We recruited patients from the Division of Gynecologic Surgery at Mayo Clinic in Rochester, MN undergoing hysterectomy by standard surgical procedures. A total of 151 patients undergoing hysterectomy were recruited. Inclusion criteria included patients who were 18 years of age or older undergoing hysterectomy for benign disease, hyperplasia, or endometrial EC. Exclusion criteria included the following: pregnancy or nursing, antibiotic treatment in the two weeks preceding surgery, and the surgeon using morcellation during the hysterectomy due to the size of the uterus or any other reason. Upon enrollment, patients completed a questionnaire on their sexual and reproductive health and history, and the answers were stored in the Redcap database for later analysis. Patients provided stool and urine specimens and vaginal, uterine, cervical, fallopian tube, and ovarian samples were procured following hysterectomy.

### Sample collection

#### Vaginal and cervical swabs

Vaginal and cervical samples were collected as described previously^22^. Briefly, vaginal swabs were collected by the surgeon, with guidance from the research team, immediately after anesthesia administration but before the standard pre-operative betadine scrub. Three sterile Dacron swabs were used to collect cells along the lateral vaginal walls. One swab was used for immediate pH measurement while the other two were placed in sterile Tris-EDTA and transported on dry ice to storage at −80 C.

#### Uterine, fallopian, and ovarian samples

Uterine, fallopian, and ovarian samples were collected as described previously^22^. Briefly, the uterus, fallopian tubes, and ovaries were transported in a sterile bag at room temperature to the pathology lab for processing under sterile conditions. The time between surgical extraction and entry into the Pathology Laboratory was under 2 minutes. After sterilization by the research team, the organs were processed at the grossing station. The pathologist’s assistant (PA) made a bilateral cut of the uterus and splayed it. The research team then collected uterine swabs (Dacron) and scrapes (sterilized pap smear spatulas) and documented sampling locations through placement of push-pins and photographs. The PA then collected samples necessary for diagnosis and the research team collected tissue biopsies from the remaining material. A petri dish of Lysogeny broth (LB) was kept open on the grossing station to detect possible contamination of the samples by airborne bacteria. Once collection was complete, the LB was swabbed and the sample placed in TE on dry ice until long-term storage at −80 C.

### Sample processing

Samples were processed as described previously^22^. Briefly, thawed swab and scrape samples were vortexed to mix any settled material and then centrifuged to pellet bacterial cells. A sterile pestle was used to macerate biopsy samples.

### DNA extraction

DNA was extracted using the MoBio PowerSoil® DNA Isolation Kit (PN 12888 Mo Bio Laboratories, Inc. Carlsbad, CA) according the manufacturer’s protocol. Approximately 100 mg of each tissue sample was used for DNA extraction. Tissue was finely minced, transferred to a Power bead tube, and homogenized for 60 seconds using MPBio Fast-prep 24. DNA concentrations were determined by Qubit dsDNA HS Assay Kit (PN Q32854 Thermo Fisher Scientific Inc., Waltham, MA). The extracted DNA was used both for rRNA gene sequencing and targeted qPCR amplification. The number of samples in each type of analysis is not exactly the same due to the failure of some samples during quality control, which resulted in their exclusion from that type of analysis. The exact number of samples for each graph and table displayed can be found in the accompanying legend.

### Dual indexing 16S PCR

A two- step PCR protocol was used to amplify the V3–V5 region of the 16S rRNA gene and then add Illumina flow cell adaptors containing indices^44^. Briefly, the primary qPCR reactions were monitored in real time using a Quant Studio 6 Flex real-time PCR system (Thermo Fisher Scientific Inc., Waltham, MA). 6 uL reactions were assembled using the following volumetric percentages: 50% DNA, 20% 5X KAPA PCR Buffer, 16.5% PCR grade water, 5% DMSO (Sigma-Aldrich, Saint Louis, MO.), 3% KAPA dNTPs (10 uM), 2% ROX (25 uM, Thermo Fisher Scientific Inc., Waltham, MA), 2% KAPA HIFI polymerase, 0.5% SYBR Green (200×, Thermo Fisher Scientific Inc., Waltham, MA), 0.5% forward primer (100 uM,) and 0.5% reverse primer (100 uM). KAPA reagents were supplied as a part of the KAPA HiFi Hot Start kit (PN KK2502, KAPA Biosystems, Woburn, MA). Sample were amplified with the following conditions: 95 °C for 5 minutes, 25 cycles of: 98 °C for 20 seconds, 55 °C for 19 seconds, and 72 °C for 60 seconds, and a final 72 °C extension for 5 minutes.

V3_357F and V5_926R primers^45^ modified with Nextera adaptors were developed in collaboration with the University of Minnesota Genomic Center in Minneapolis, MN.

V3_341F_Nextera:

TCGTCGGCAGCGTCAGATGTGTATAAGAGACAGCCTACGGGAGGCAGCAG

V5_926R_Nextera:

GTCTCGTGGGCTCGGAGATGTGTATAAGAGACAGCCGTCAATTCMTTTRAGT

Primary PCR products were diluted 1:100 in PCR grade water for secondary PCR reactions. 10 uL reactions were assembled using the following volumetric percentages: 50% DNA, 20% 5X KAPA PCR Buffer, 5% DMSO (Sigma-Aldrich, Saint Louis, MO.), 3% KAPA dNTPs (10 uM), 2% KAPA HIFI polymerase, 10% forward primer (5 uM,) and 10% reverse primer (5 uM). KAPA reagents were supplied as a part of the KAPA HiFi Hot Start kit (PN KK2502, KAPA Biosystems, Woburn, MA). PCR cycling conditions were 95 °C for 5 minutes, 10 cycles of: 98 °C for 20 seconds, 55 °C for 15 seconds, and 72 °C for 60 seconds, and a final 72 °C extension for 5 minutes.

The following indexing primer design was utilized^44^ (X indicates the position of the indices).

Forward i5 primer: AATGATACGGCGACCACCGAGATCTACACXXXXXXXXTCGTCGGCAGCGTC

Reverse i7 primer: CAAGCAGAAGACGGCATACGAGATXXXXXXXXGTCTCGTGGGCTCGG

### Normalization and pooling of 16S libraries

PCR products were diluted to 20 uL with PCR grade water and cleaned up using 1.0X AMPureAP beads (Beckman Coulter, Brea, CA), vacuum-dried, reconstituted in 12 uL of PCR grade water, quantified using a Quant-It dsDNA HS assay kit (Thermo Fisher Scientific Inc., Waltham, MA), normalized and pooled. The sequencing pool was concentrated, cleaned up using 1.8X AMPureAP beads (Beckman Coulter, Brea, CA) quantified using a Quant-It dsDNA HS assay kit (Thermo Fisher Scientific Inc., Waltham, MA). Sequence pool was assessed for purity and the presence of 725 bp peak (±20%) using a 2200 TapeStation system and D1000 Screen tape/reagents (Agilent Technologies, Santa Clara, CA).

### Sequencing

The 16S amplicon pools are quantified using the KAPA SYBR FAST qPCR kit (KAPA Biosystems, Woburn, MA), diluted to 2 nM, denatured with an equal volume of 0.2 N NaOH, diluted to 6 pM with Illumina HT1 buffer, spiked with 30% PhiX, heat denatured at 96 °C for 5 minutes and sequenced using the MiSeq 600 cycle v3 kit (Illumina, San Diego, CA) and MCS v2.6.1.

### Pipeline for processing of 16S data

The sequence reads were processed via the *IM TORNADO* bioinformatics pipeline^46^ into operational taxonomic units (OTUs) at 97% similarity level. OTUs were assigned taxonomy using the RDP classifier trained on the GreenGenes database (v13.5)^47^. A phylogenetic tree based on FastTree algorithm was constructed based on the OTU representative sequences^48^. Reads from replicate samples from the same patient and location were aggregated. Singleton OTUs were removed as well as samples with depth less than 500 as a quality control step. Detailed information can be found in Supplemental Table S12.

### Sequencing data analysis

We used custom scripts (available at Figshare) written in the statistical language R^49^ for statistical analysis. Significant differences among patient clinical characteristics were determined using two-tailed Student’s t test or chi-squared test where appropriate. For the sequencing data, we first split the patients by primary pathology (non-EC, EC, or hyperplasia) and analyzed organ microbiomes of each group. Multiple samples per location were aggregated per patient prior to rarefaction. Sequencing read rarefaction is indicated in the figures. Due to the low number of patients in the hyperplasia group (N = 7) these samples were excluded from the rest of the analysis. We eliminated ovary and fallopian tube samples from the remainder of the analysis due to low reads and inconsistent sampling per patient. Supplemental Fig. S8 shows significant differences between patient samples and sequencing controls (sampling, PCR, and positive controls).

#### α and β diversity

α and β diversity are standard ecological measures of microbial diversity representing, respectively, the number of unique taxa per sample and similarity in composition between samples^50^. We calculated the observed number of OTUs as the α diversity measure for each organ within the non-EC and EC groups after rarefaction (Supplemental Fig. S9). We also calculated the Shannon index as our main α-diversity metric, which was generally concordant with observed number of OTUs. We then fitted a linear model for independent samples (“lm” function in R “stats” package) and adjusted for covariates when necessary. The t-type was used to determine statistical significance. For β diversity, we rarefied the data prior to calculating the various distance measures. To test the association between the covariates and β-diversity measures, we used PERMANOVA, a distance-based analysis of variance method based on permutation (999 permutations, “adonis” function in the R “vegan” package 1.17–4^51^). An omnibus test, which is a permutation test taking the minimum of the P-values of individual β-diversity measures as the test statistic, was used to combine multiple sources of association evidence provided by different β-diversity measures and an overall association P-value was reported (“PermanovaG” function in the R “GUniFrac” package v1.1^23^). Ordination plots were generated using classic multi-dimensional scaling (MDS) as implemented in R (“cmdscale” function in the R “stats” package). Supplemental Table S13 contains degrees of freedom and F values for all PERMANOVA tests.

#### Differential abundance analysis

Based on the estimated abundance changes in our prior study with approximately 30 patients^22^, the sample size in this study offers sufficient power to detect changes at a false discovery rate of 10% using the online power calculator “shinyMB” (https://fedematt.shinyapps.io/shinyMB/). We analyzed differential abundance at the phylum, family, genus, and species levels, filtering rare taxa prevalent at less than 10% of samples or taxa with a maximum proportion (relative abundance) less than 0.2% to reduce the number of necessary tests. We fit a generalized linear model with over-dispersed Poisson distribution to the count data. We estimated the library sizes (sequencing depth) using the Geometric Mean of Paired Rations (GMPR) normalization method^52^ and the log of the GMPR size factors were used in the Poisson model as an offset to account for variable library sizes. The data was winsorized (97% upper quantile) to reduce the possible impact of outliers on parameter estimates before fitting the model. To improve our power in detecting differential taxa, we pooled the cervix and vagina data, which demonstrated consistent changes between them. We assessed statistical significance using the Wald test and used a false discovery rate (FDR) control (B-H procedure, “p.adjust” in R “stats” package) to correct for multiple testing. FDR-adjusted p values (q values) less than 0.05 were considered significant.

#### Organ correlation

A permutation test based on the Bray-Curtis distance was used to test for correlation between organs. The test statistic was calculated as the distance between organs from different participants minus the distance between organs from the same participant. A block-permutation scheme was used to retain within-subject correlation. Samples from the same organ site and the same patient were assigned a different subject ID. We used 1,000 permutations to assess significance. We calculated p values as the percentage of permutations that produce a test statistic more extreme than what was observed.

All statistical analyses were performed in R 3.3.1 (R Development Core Team).

#### Network visualization

A network characterizing the statistically-significant associations between EC-associated OTUs and their risk factors, and the correlations between OTUs, was visualized using Cytoscape v3.7.1. Edge weights were set according to the strength of association or correlation. Genus- or species-level annotation for each OTU was determined by identifying its phylogenetically closest bacterial genome in the GreenGenes database (v13.5).

### Detection of enriched bacteria by qPCR

#### Bacterial strains

*Porphyromonas somerae* (ATCC BAA-1230) and *Atopobium vaginae* (ATCC BAA-55) were obtained in freeze-dried form and propagated in Thio Remel broth (ThermoFisher Scientific, Waltham, MA) in an anaerobic environment (COY Rigid Anaerobic Chamber; Coy Laboratory Products Inc, Grass Lake, MI) with the following atmosphere: nitrogen 80%, carbon dioxide 10%, and hydrogen 10%. They were subsequently plated on Trypticase soy agar (TSA) with 5% sheep blood (ThermoFisher Scientific, Waltham, MA) (following ATCC guidelines). The plates were placed in the anaerobic environment for 48 hours before use to allow for reduction of oxygen from the media. The microbes were incubated at 37 °C in an anaerobic environment for 48 hours for *P. somerae* and 72 hours for *A. vaginae*. We also produced cultures of *Escherichia coli K12 Strain MSG 123* (ATCC PTA-7555), *Staphylococcus aureus* (ATCC 12600), and *Lactobacillus iners* (ATCC 55195) to test PCR probe specificity.

#### Bacterial DNA extraction

The bacterial cells from each strain were harvested from the broth solution. Microbial DNA was extracted using BiOstic Bacteremia DNA Isolation Kit (MoBio Laboratories, Inc., Carlsbad, CA), according to the manufacturer’s instructions. Microbial DNA was quantified using Qubit Fluorometric quantitation (ThermoFisher Scientific, Waltham, MA). The resulting DNA was stored at −80 °C.

#### Design of qPCR primers and probes

Specific 16S rDNA primers and probes for *P. somerae* and *A. vaginae* were designed using GenScript Real-time PCR (Taqman) Primer Design online software (https://www.genscript.com/tools/real-time-pcr-tagman-primer-design-tool). The primers and probes were screened for self-dimers, hetero-dimers, hair-pin configurations, melting temperatures, and percent of GC values using OligoAnalyzer online software (Integrated DNA Technologies, https://www.idtdna.com/calc/analyzer). Each primer and probe were compared to the National Center for Biotechnology Information (NCBI) database through Basic Local Alignment Search Tool (BLAST) program (https://blast.ncbi.nlm.nih.gov/Blast.cgi).

The sequences for the primer-probe combinations for universal, *P somerae*, and *A vaginae* are listed in Supplemental Table S14. The Eubacteria primer-probe sequences were obtained from previous studies^53^. The oligonucleotide probes for *P somerae* and *Eubacteria* were labeled with fluorescent reporter dye 6-carboxyfluorescein (6-FAM) at the 5′ end with an internal ZEN Quencher and 3′ Iowa Black Fluorescent Quencher. The *A vaginae* oligonucleotide probes were labeled with fluorescent reporter dye NED at the 5′ end and the dual quencher with internal ZEN and 3′ Iowa Black FQ (Integrated DNA Technologies; Coralville, IA). The primers and probes were optimized to determine the minimum concentration to yield the highest amplification efficiency with the lowest threshold cycle (Ct) by performing polymerase chain reaction (PCR) assays in triplicate with multiple differing concentrations of primers and probes.

#### qPCR limit of detection

The limits of detection for each microbe with real-time PCR was determined using serial 10-fold dilutions of pure genomic DNA from the cultured *P. somerae* (1 ng/µL to 1 × 10^−6^ ng/µL) and *A. vaginae* (1.8 ng/µL to 1.8 × 10^−7^ ng/µL). Vaginal swabs from the initial cohort of 31 patients whose sequencing data was published in our pilot study^22^ were used as a confirmatory test to determine if the limits of detection changed in a real patient sample with multiple microbes present. Specificity for each real-time PCR assay was determined by using microbial DNA harvested from pure cultures of different microbes, including *Escherichia coli*, *Staphylococcus aureus*, and *Lactobacillus iners*, as well as cross-usage of the *P. somerae* and *A. vaginae*.

#### Amplification

The microbial 16S rDNA was amplified through PCR in the following working solution: 10 µL of 2x Taqman Universal Master Mix II (AmpliTaq Gold DNA polymerase, deoxynucleotide triphosphates, ROX Passive Reference dye, optimized buffer components; ThermoFisher Scientific, Grand Island, NY), 2 µL (9 µM) forward primer, 2 µL (9 µM) reverse primer, 2 µL (2.5 µM) probe, 2 µL sample DNA, and 2 µL molecular grade water up to a final volume of 50 µL per reaction. The qPCR standard universal thermal cycling protocol was performed using the QuantStudio 6 Flex (ThermoFisher Scientific) as follows: activation of DNA polymerase at 50 °C for 2 minutes then increased to 95 °C for 10 minutes followed by 40 cycles of denaturation at 95 °C for 15 seconds followed by annealing/extension phase at 60 °C for 1 minute. Data collection occurred during the annealing/extension phase. Samples were plated in 384-well plates and covered with optical adhesive plastic. Plates were centrifuged at 2000 RPM for 2 minutes to remove bubbles from bottom of wells. All samples were run in triplicate with positive and negative controls.

#### Blind analysis of extended cohort

Based on the sequencing data as well as our previously published results^22^, we investigated the potential for particular microorganisms in the vaginal microbiome (*P somerae* and *A vaginae*) and high pH (>4.5) to be used as surrogate indicators of EC. Each patient sample was given a random number by a team member; the researcher performing the test was blinded to each patient’s diagnosis. Each test was done in triplicate and included the simultaneous use of *P somerae*, *A vaginae*, and universal probes, along with a negative control (water only). A test was only considered valid if all 3 replicates of the universal probe were positive, all 3 replicates of the negative control were negative, and all 3 replicates of the test probe provided a consistent result. All replicates provided a consistent result for all samples in our study. A representative qPCR result for a test sample is shown in Supplemental Fig. S10.

#### Statistical analysis of qPCR data

Diagnosis of EC vs non-EC controls was modeled using logistic regression. Individual predictors were tested using likelihood ratio tests. Because of the relatively small sample size and sparseness of several predictor variables, tests of interactions of the well-known risk factors (obesity and menopausal status) and other predictors were not conducted (assuming lack of adequate statistical power to detect interactions); however, we analyzed subsets of women considered to be at high risk. A multipredictor logistic regression model also was fit, in which each predictor was tested with a Wald test adjusting for all other predictors. Sensitivity, specificity, positive predictive value, and area under the receiver operating characteristic curve (plus 95% CIs) were calculated for the predictive ability of individual risk factors and EC diagnosis. We analyzed the association of risk factors among patients without EC to assess how these factors relate in the absence of EC. To test for association with a binary predictor, a likelihood ratio test was used based on a logistic model. To test for association between continuous predictors, a *t* test of the Pearson correlation coefficient was used. A *P* value less than 0.05 was considered statistically significant. All analyses were conducted using R v 3.2.3.

### Ethics approval and consent to participate

The Mayo Clinic Institutional Review Board approved 2 protocols: #12-004445 to perform sequencing and for patient enrollment with written consent being provided by all patients, and #15-000842 for the use of patient samples to develop the qPCR assay.

### Consent for publication

Consent was obtained from study participants; however, all patient data in the study has been de-identified.

## Supplementary information

Supplemental Data

Supplemental Table S11

## Data Availability

R code and OTU table and patient metadata can be found at Figshare (Metadata: 10.6084/m9.figshare.6837893, OTU table: 10.6084/m9.figshare.6837890, R functions: 10.6084/m9.figshare.6817103, R analysis code: 10.6084/m9.figshare.6809978). Raw sequencing files can be found on the SRA database (BioProject accession PRJNA481576).
