# Supplementary material for: Postmenopause as a key factor in the composition of the Endometrial Cancer Microbiome (ECbiome)
Source: Sci Rep. 2019 Dec 16;9:19213. doi: 10.1038/s41598-019-55720-8 (PMC6915778; doi:10.1038/s41598-019-55720-8)
Supplement: Supplementary file 1 — Supplemental Data [file 41598_2019_55720_MOESM1_ESM.pdf]

Supplemental Data

Postmenopause as a key factor in the composition of the Endometrial Cancer Microbiome (ECbiome)

Authors

Dana M. Walsh[1,2§], Alexis N. Hokenstad[3§], Jun Chen[1,4], Jaeyun Sung[1,2,5,6], Gregory D. Jenkins[4], Nicholas Chia[1,2], Heidi Nelson[1,2], Andrea Mariani[7]*, Marina R. S. Walther-Antonio[1,2,3]*

[1]Microbiome Program, Center for Individualized Medicine, Mayo Clinic, Rochester, Minnesota, USA, [2]Division of Surgical Research, Department of Surgery, Mayo Clinic, Rochester, Minnesota, USA, [3]Department of Obstetrics & Gynecology, Mayo Clinic, Rochester, Minnesota, USA, [4]Department of Health Sciences Research, Mayo Clinic, Rochester, Minnesota, USA, [5]Division of Rheumatology, Department of Internal Medicine, Mayo Clinic, Rochester, Minnesota, USA, [6]Department of Molecular Pharmacology & Experimental Therapeutics, Mayo Clinic, Rochester, MN, USA, [7] Division of Gynecologic Surgery, Mayo Clinic, Rochester, MN, USA

[§]These authors contributed equally to this work.

*Corresponding authors:

Andrea Mariani (mariani.andrea@mayo.edu)

Marina R. S. Walther-Antonio (waltherantonio.marina@mayo.edu)

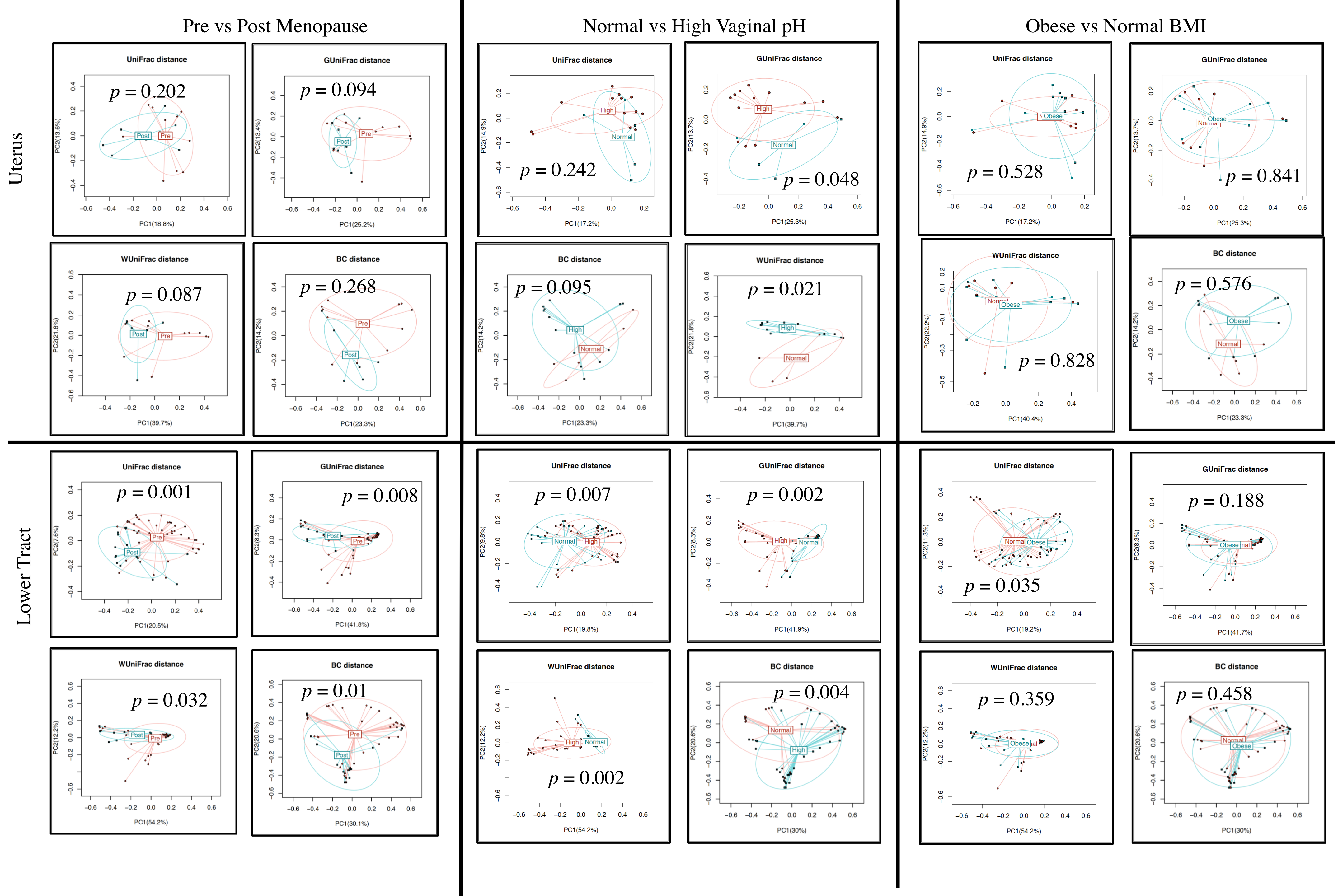

Supplemental Figure S1. Additional β diversity tests for significant differences among cancer risk factors in benign patients. PERMANOVA tests for each patient factor were adjusted for the remaining two factors (menopause status, obesity, vaginal pH).

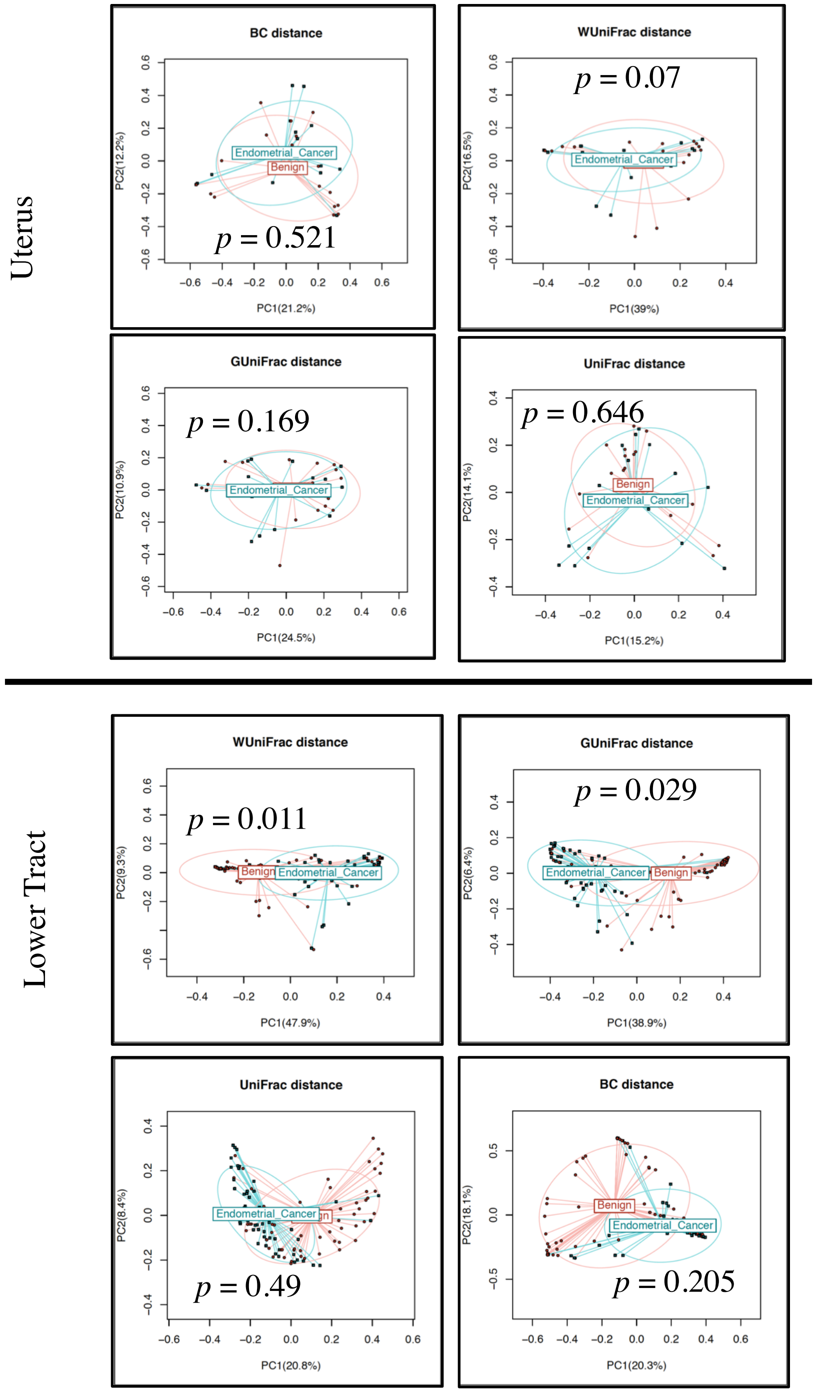

Supplemental Figure S2. Additional β diversity tests for significant differences in the uterus and lower tract between patients with and without cancer. PERMANOVA tests for each patient factor were adjusted for the remaining two factors (menopause status, obesity, vaginal pH).

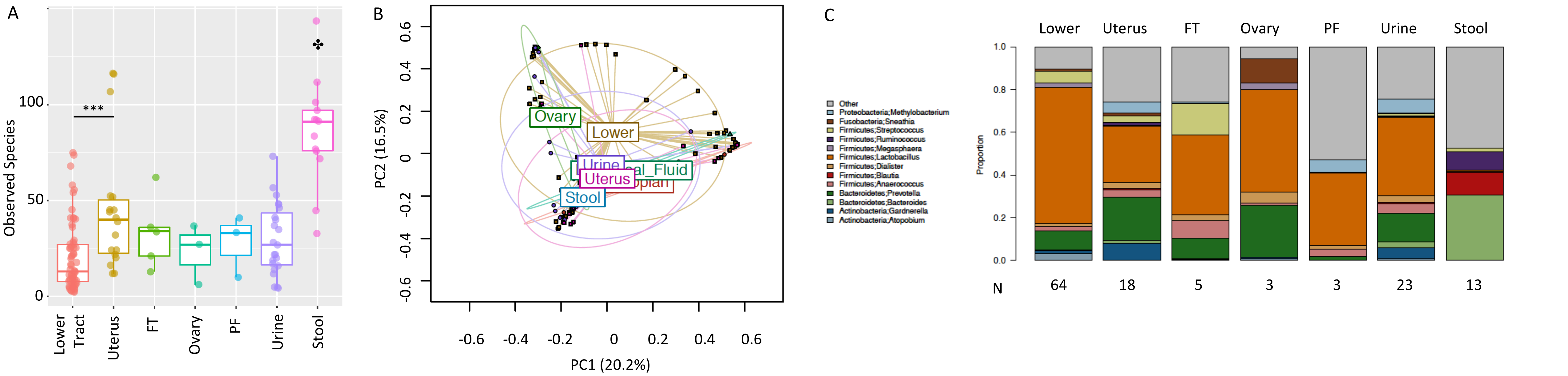

Supplemental Figure S3. Bacterial community diversity within the reproductive tract, peritoneal fluid, urine, and stool of women with benign hysterectomies. (A) α-diversity (observed number of species) per site, (B) β-diversity (Bray-Curtis distance) of these samples by site and (C) Microbial composition per site. Samples were rarefied prior to diversity analyses. FT = fallopian tube, PF = peritoneal fluid. Lower Tract N = 64, Uterus N = 18, FT N = 5, Ovary N = 3, PF N = 3, Urine N = 23, Stool N = 13. ✤ = Stool is significantly different from all organs. 'Other' encompasses taxa < 5% of total proportion.

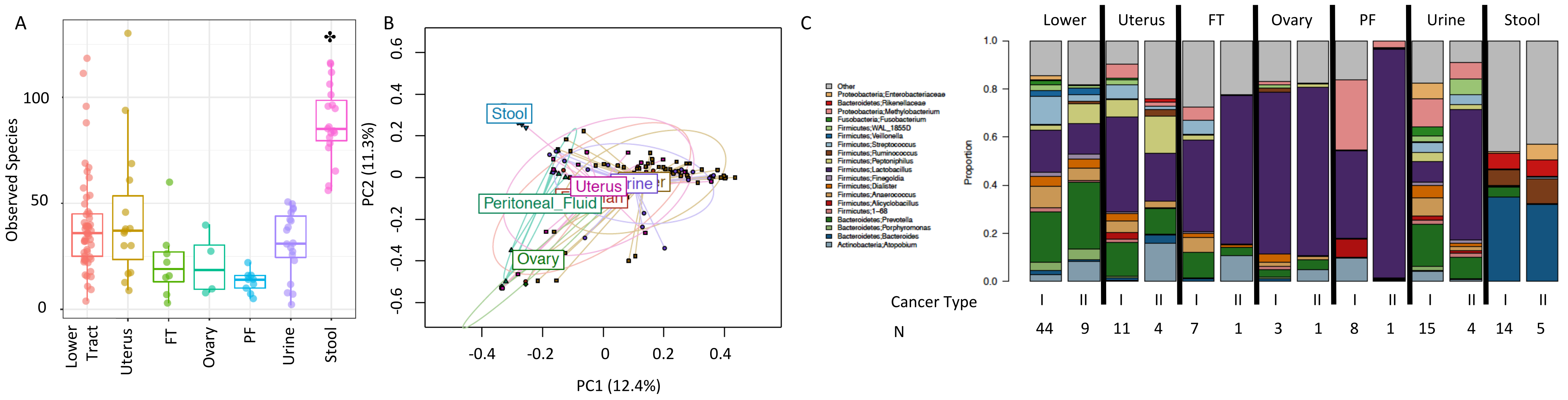

Supplemental Figure S4. Bacterial community diversity within the reproductive tract, urine, and stool of women with endometrial cancer. (A) α-diversity (observed number of species) per site. (B) β-diversity (Bray-Curtis distance) of these samples by site. (C) Proportion of each genus by site and cancer type. Samples were rarefied prior to diversity analysis. Type I cancer: Fallopian N = 7, Lower N = 44, Ovary N = 3, Peritoneal Fluid N = 8, Stool N = 14, Urine N = 15, Uterus N = 11. Type II cancer: Fallopian N = 1, Lower N = 9, Ovary N = 1, Peritoneal Fluid N = 1, Stool N = 5, Urine N = 4, Uterus N = 4. FT = Fallopian Tube, PF = Peritoneal Fluid, I = Type I Endometrial Cancer, II = Type II Endometrial Cancer. ✤ = Stool is significantly different from all organs. Lower tract and uterus alpha diversity are significantly different from peritoneal fluid alpha diversity. 'Other' encompasses taxa < 5% of total proportion.

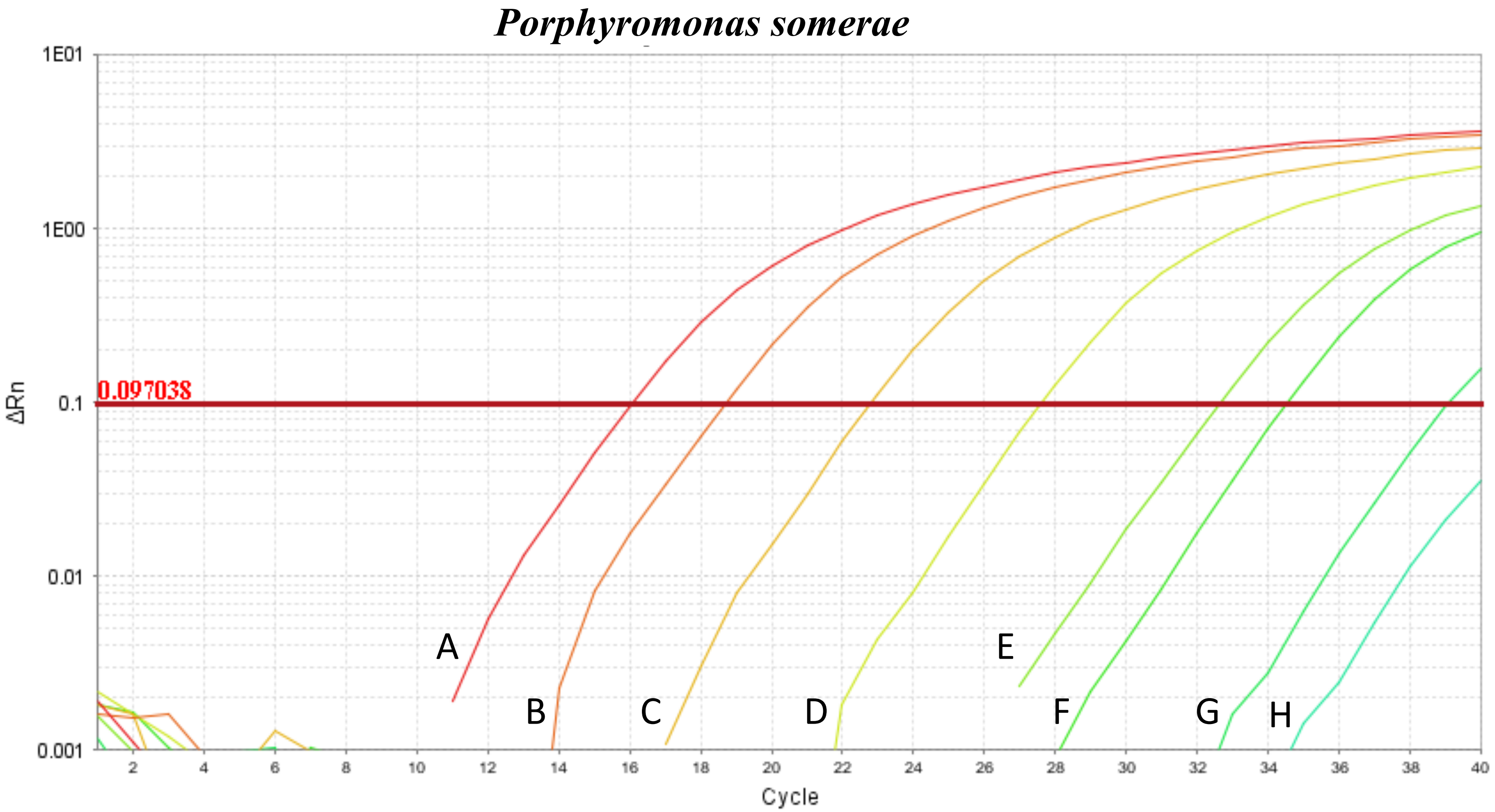

Supplemental Figure S5. Serial 10 fold dilutions of *P. somerae*. ΔRn, change in fluorescence activity. Concentration of DNA as follows: A-10ng/μL, B-1ng/μL, C-1x10^{-1}ng/μL, D-1x10^{-2}ng/μL, E-1x10^{-3}ng/μL, F-1x10^{-4}ng/μL, G-1x10^{-5}ng/μL, H-1x10^{-6}ng/μL. A concentration of 1x10^{-7}ng/μL did not amplify, neither did the negative control (H_2O only).

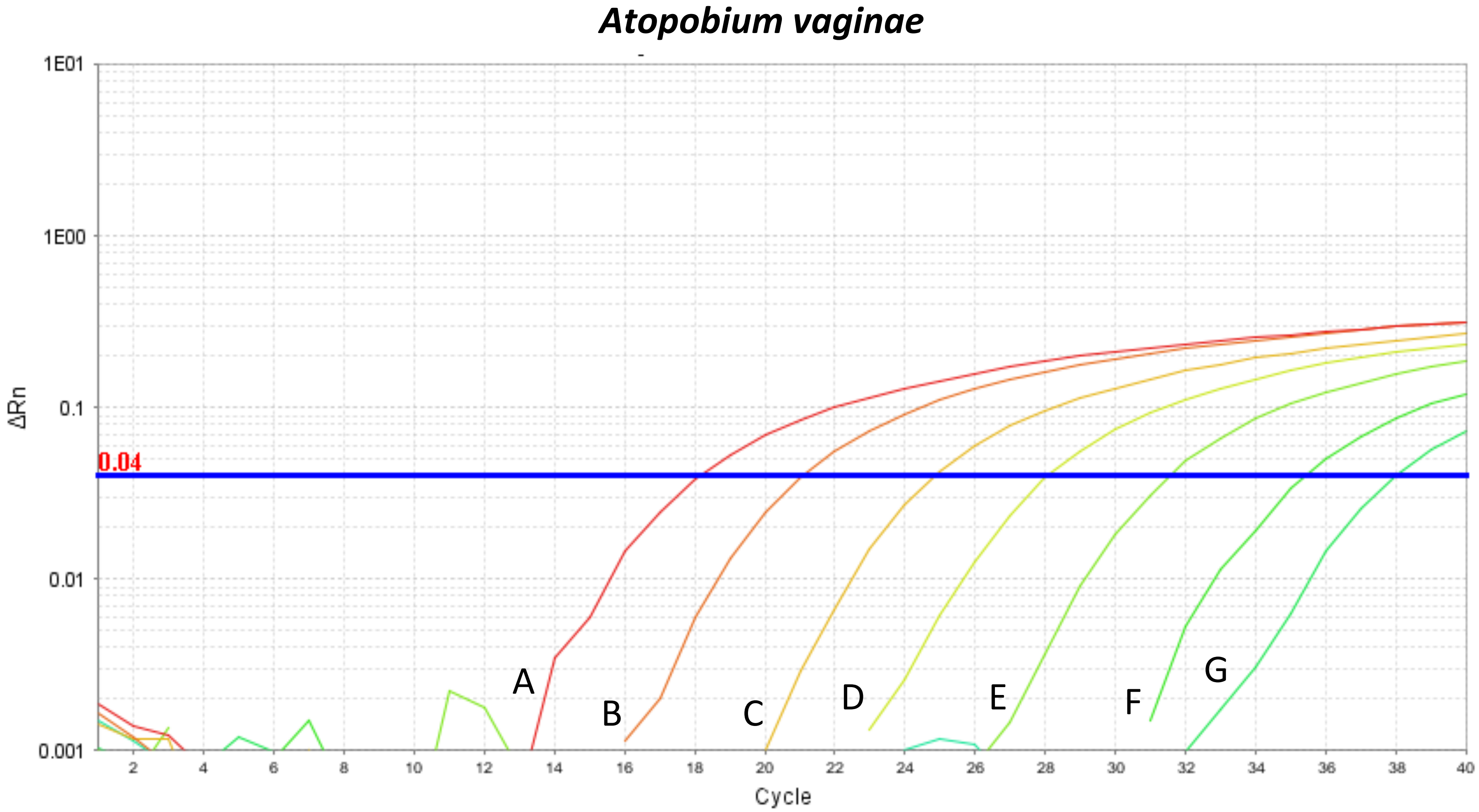

Supplemental Figure S6. Serial 10 fold dilutions of *A. vaginae*. ΔRn, change in fluorescence activity. Concentration of DNA as follows: A-1.8ng/µL, B-1.8x10^{-1}ng/µL, C-1.8x10^{-2}ng/µL, D-1.8x10^{-3}ng/µL, E-1.8x10^{-4}ng/µL, F-1.8x10^{-5}ng/µL, G-1.8x10^{-6}ng/µL. A concentration of 1x10^{-7}ng/µL did not amplify, neither did the negative control (H_2O only).

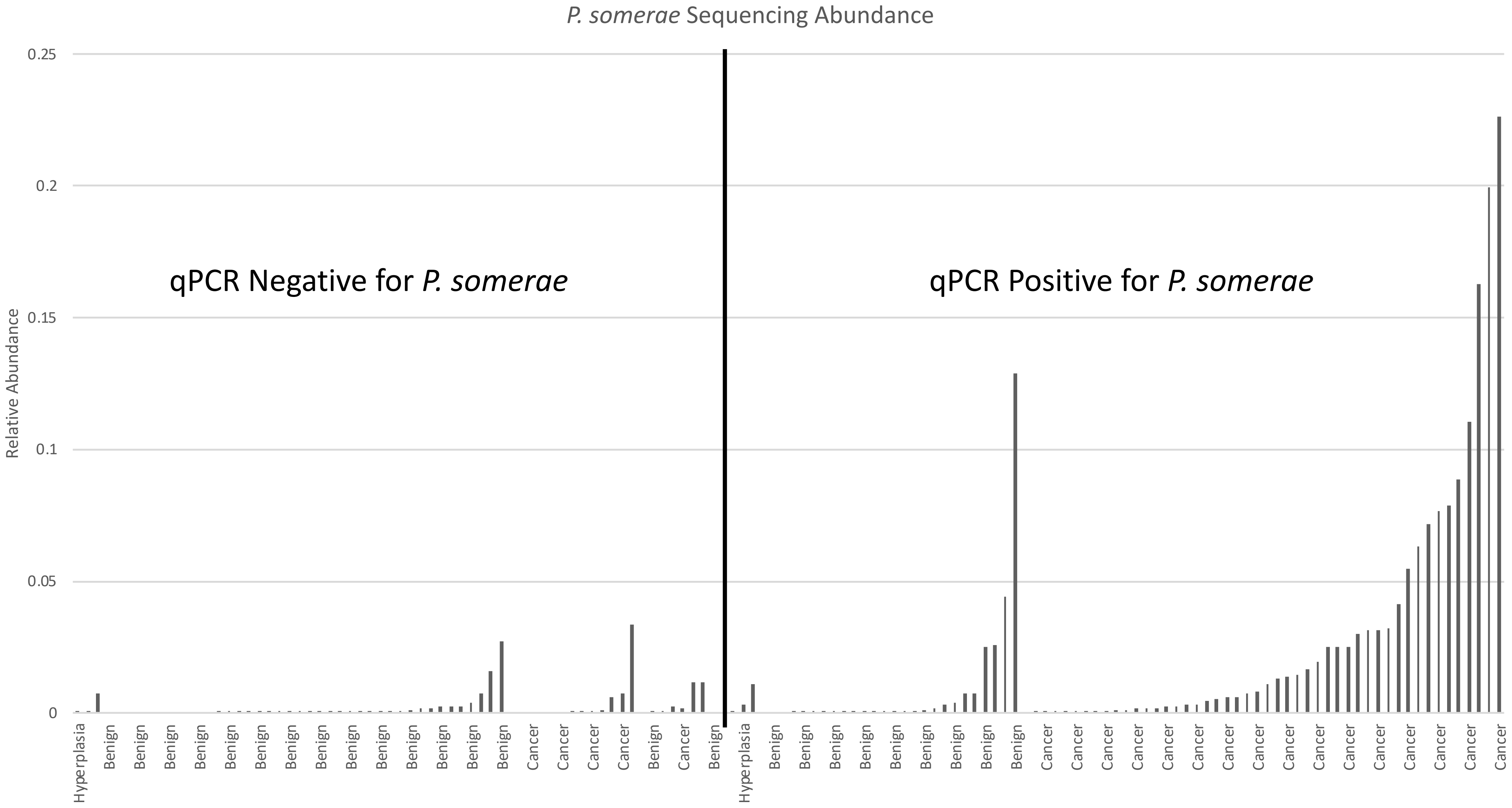

Supplemental Figure S7. Comparison of qPCR results with *P. somerae* sequencing relative abundance according to patient pathology.

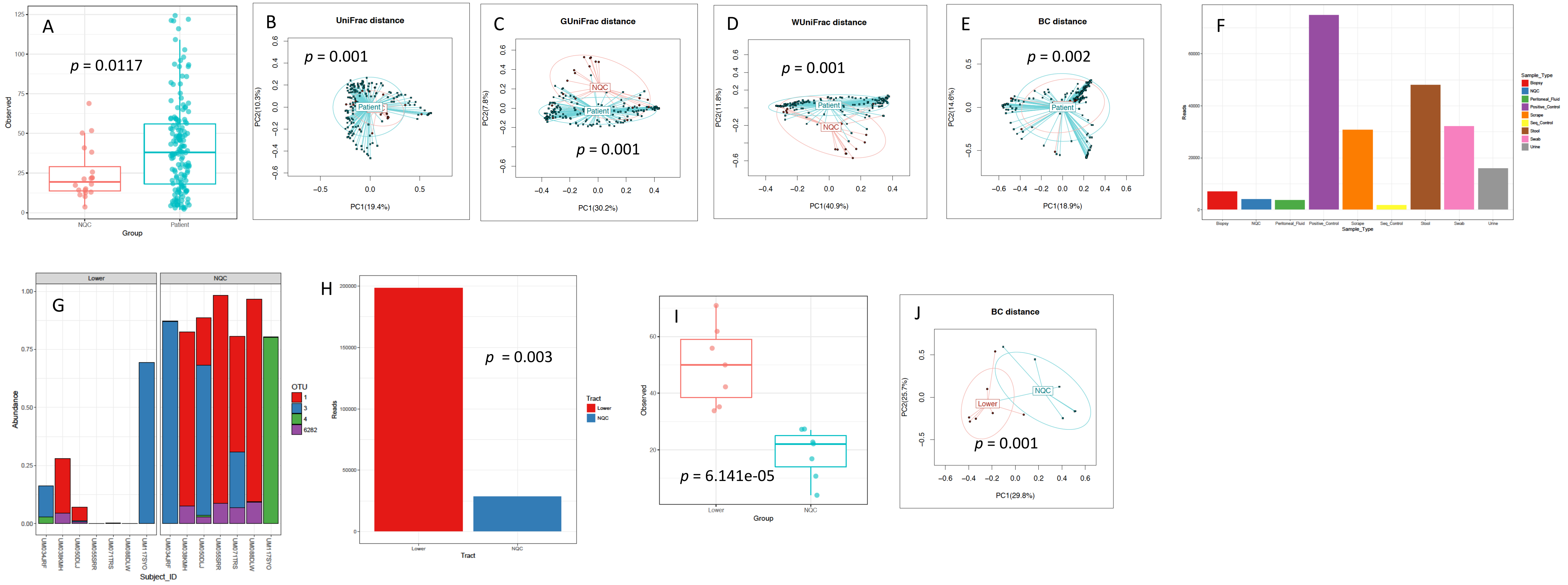

Supplemental Figure S8. Controls (sampling, PCR amplification, and positive) are significantly different from patient samples. Negative sampling controls (NQC) are significantly different from their matched patient samples based on (A) α-diversity (observed number of species) and four different β-diversity metrics: (B) unweighted UniFrac distance, (C) generalized UniFrac distance, (D) weighted UniFrac distance, and (E) Bray-Curtis distance. (F) mean sequence read for each sample type, including positive controls. Seven out of twenty sampling controls contained high lactobacillus abundance; these are compared to their matched patient controls (Lower) in (G). These samples are significantly different from the negative controls by number of reads (H, paired Student's T test), alpha diversity (I, observed number of species) and beta diversity (J, Bray-Curtis distance).

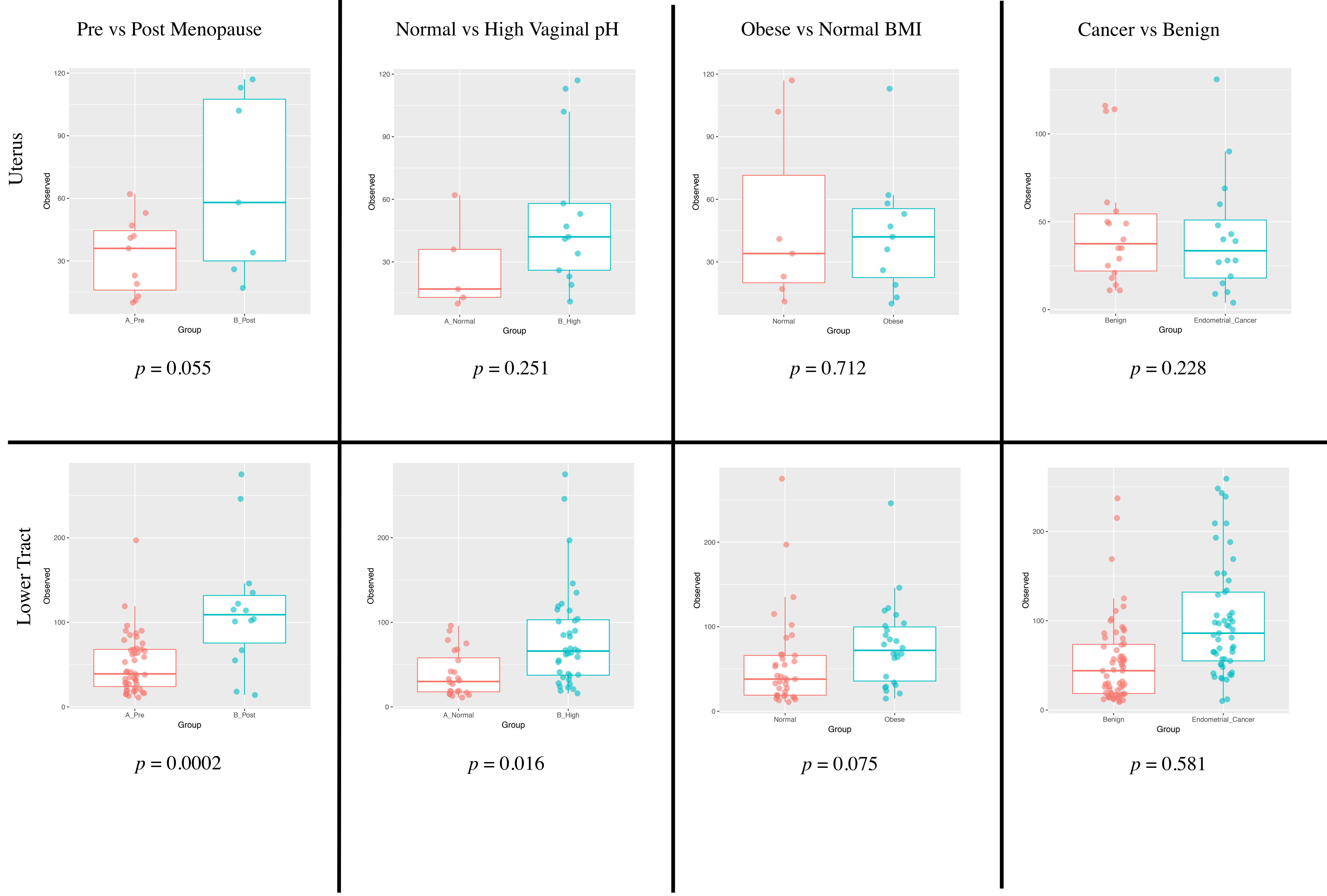

Supplemental Figure S9. Observed OTUs α-diversity for menopause status, vaginal pH, and obesity in patients without cancer in the lower tract and uterus and for the lower tract and uterus in patients with and without cancer.

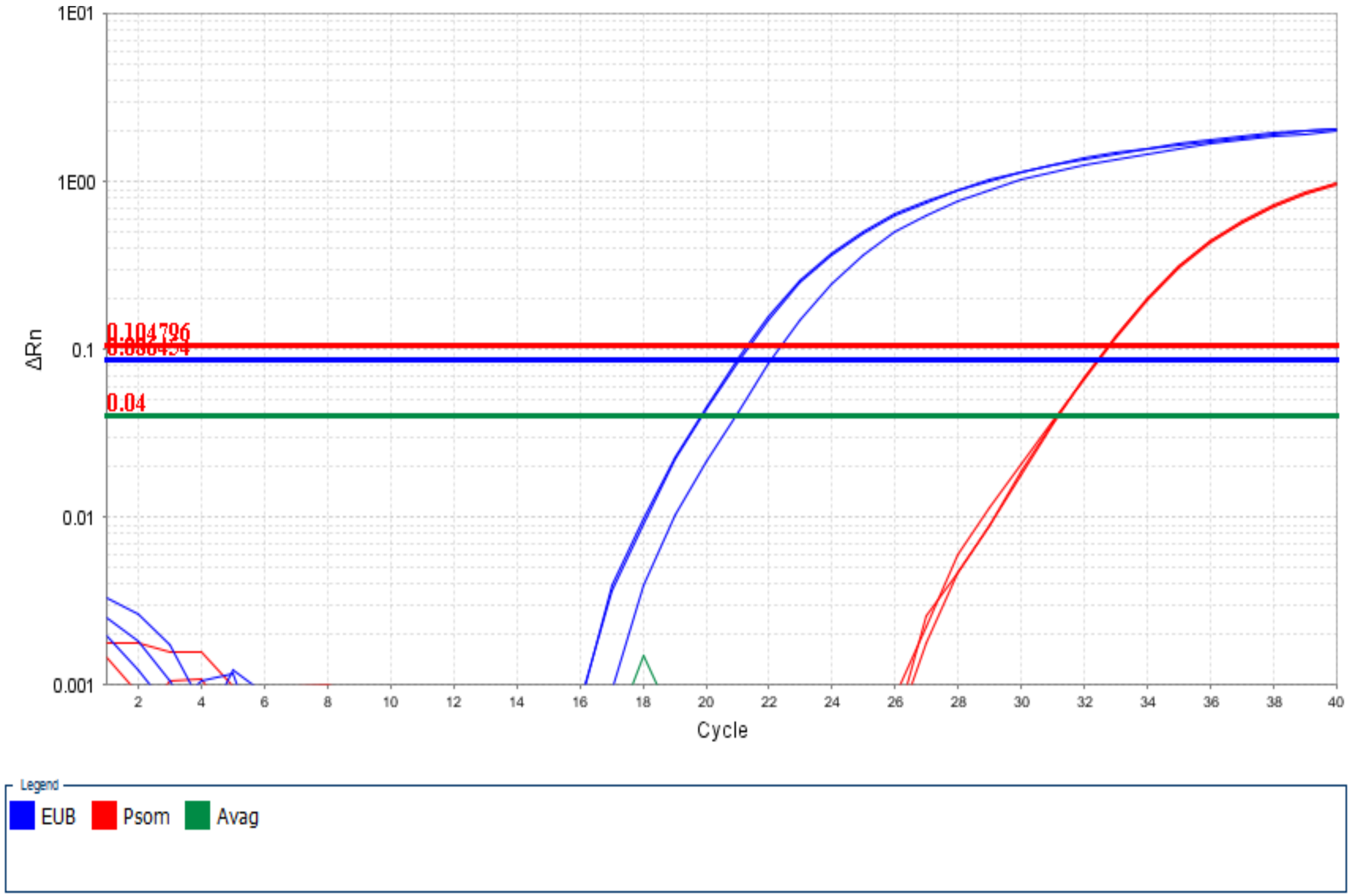

Supplemental Figure S10. Example of a sample qPCR test positive for the presence of *P. somerae* and negative for the presence of *A. vaginae*. All tests were done in triplicate. Only samples with positive identification of bacteria with the universal bacteria probe were considered valid.

Supplemental Table S1. OTUs Enriched in Postmenopausal Women at Q < 0.1

OTU	P Value	Q Value	Effect Size (2.5%, 97.5%)
OTU96: Actinobacteria; *Tessaracoccus*	2.740E-05	0.001	2.667 (1.421, 3.914)
OTU88: Actinobacteria; *Atopobium*	1.570E-05	0.001	3.555 (1.942, 5.168)
OTU15701: Firmicutes; *Anaerococcus*	1.075E-04	0.002	1.814 (0.896, 2.732)
OTU331: Bacteroidetes; *Porphyromonas*	1.047E-04	0.002	3.356 (1.66, 5.051)
OTU332: Actinobacteria; *Luteococcus*	8.590E-05	0.002	3.048 (1.527, 4.569)
OTU217: Firmicutes; *Gallicola*	2.289E-04	0.004	1.52 (0.712, 2.329)
OTU41: Firmicutes; *1-68*	2.392E-04	0.004	2.531 (1.181, 3.882)
OTU32: Firmicutes; *Peptoniphilus*	0.001	0.015	0.859 (0.342, 1.376)
OTU58: Proteobacteria; *Campylobacter*	0.001	0.016	1.728 (0.671, 2.786)
OTU33: Firmicutes; *1-68*	0.002	0.020	1.825 (0.675, 2.975)
OTU13179: Firmicutes; *Anaerococcus*	0.002	0.021	1.089 (0.39, 1.787)
OTU15196: Actinobacteria; *Varibaculum*	0.002	0.021	3.029 (1.076, 4.983)
OTU22: Bacteroidetes; *Bacteroides*	0.003	0.025	2.594 (0.865, 4.323)
OTU38: Firmicutes; *Dialister*	0.003	0.025	1.44 (0.487, 2.392)
OTU274: Firmicutes; *Clostridium*	0.004	0.027	2.33 (0.73, 3.91)
OTU4369: Firmicutes; *Anaerococcus*	0.005	0.031	1.017 (0.311, 1.724)
OTU190: Firmicutes; *Tepidimicrobium*	0.005	0.032	2.061 (0.615, 3.508)
OTU16: Firmicutes; *Anaerococcus*	0.008	0.043	0.911 (0.243, 1.58)
OTU207: Actinobacteria; *Alloscardovia*	0.008	0.043	2.282 (0.601, 3.963)
OTU39: Firmicutes; *Peptoniphilus*	0.013	0.061	1.22 (0.261, 2.178)
OTU22441: Firmicutes; *Faecalibacterium*	0.013	0.061	2.082 (0.445, 3.719)
OTU43: Bacteroidetes; *Porphyromonas*	0.012	0.061	1.994 (0.435, 3.552)
OTU105: Firmicutes; *Peptoniphilus*	0.017	0.075	1.023 (0.182, 1.865)
OTU24: Bacteroidetes; *Bacteroides*	0.018	0.075	2.206 (0.281, 4.03)
OTU118: Actinobacteria; *Actinomyces*	0.018	0.075	1.369 (0.237, 2.501)
OTU4476: Firmicutes; *Moryella*	0.019	0.075	0.847 (0.142, 1.553)
OTU56: Firmicutes; *Blautia*	0.022	0.085	1.853 (0.27, 3.436)
OTU231: Firmicutes; *Peptococcus*	0.023	0.086	1.565 (0.216, 2.914)

Supplemental Table S2. P and Q Values for EC-Associated Taxa. Taxa with Q values < 0.1 were identified among those enriched in endometrial cancer. OTUs with positive effect sizes are enriched in cancer while those with negative effect sizes are enriched in patients without cancer. OTUs in bold are enriched only in cancer. *Enriched in cancer and postmenopause (Q values in **Supplemental Table S1**).

	**Endometrial Cancer**		
OTU	P Value	Q Value	Effect Size (2.5%, 97.5%)
**OTU28: Bacteroidetes; *Porphyromonas***	5.730E-06	**8.647E-04**	2.429 (1.38, 3.478)
OTU105: Firmicutes; *Peptoniphilus**	7.909E-04	**0.032**	0.935 (0.389, 1.481)
**OTU99: Firmicutes; *Anaerococcus***	0.001	**0.032**	1.121 (0.438, 1.403)
OTU13179: Firmicutes; *Anaerococcus**	6.423E-04	**0.032**	1.044 (0.444, 1.643)
OTU32: Firmicutes; *Peptoniphilus**	0.001	**0.032**	1.078 (0.43, 1.725)
**OTU268: Firmicutes; *Peptoniphilus***	0.001	**0.032**	2.122 (0.834, 3.411)
OTU231: Firmicutes; *Peptococcus**	0.002	**0.047**	1.404 (0.506, 2.303)
OTU58: Proteobacteria; *Campylobacter**	0.006	**0.055**	1.064 (0.21, 1.812)
**OTU236: Actinobacteria; *Arcanobacterium***	0.006	**0.055**	0.961 (0.279, 1.643)
**OTU19801: Actinobacteria; *Varibaculum***	0.006	**0.055**	0.987(0.281, 1.694)
**OTU64: Fusobacteria; *Fusobacterium***	0.004	**0.055**	1.451 (0.462, 2.44)
OTU5651: Firmicutes; *Lactobacillus*	0.004	**0.055**	-1.589 (-2.684, -0.493)
OTU217: Firmicutes; *Gallicola**	0.004	**0.055**	1.211 (0.284, 2.04)
**OTU29: Firmicutes; *Finegoldia***	0.006	**0.055**	0.888 (0.257, 1.519)
OTU6282: Firmicutes; *Lactobacillus*	0.005	**0.055**	-1.54 (-2.613, -0.467)
OTU118: Actinobacteria; *Actinomyces**	0.003	**0.055**	1.165 (0.29, 1.94)
**OTU40: Bacteroidetes; *Prevotella***	0.004	**0.055**	0.803 (0.252, 1.355)
OTU16: Firmicutes; *Anaerococcus**	0.008	**0.066**	0.77 (0.202, 1.337)
**OTU13: Firmicutes; *Anaerococcus***	0.011	**0.084**	0.906 (0.211, 1.6)

Supplemental Table S3. OTUs with Q <0.1 in Endometrial Cancer BLAST Species Matches

OTU Number	Genus	Species	Strain	BLAST % Match	E Value
28	*Porphyromonas*	*somerae*	JCM 13867 & DSM 23386	100	7e-147
105	*Peptoniphilus*	*coxii*	RMA 16757	100	7e-147
99	*Anaerococcus*	*vaginalis*	JCM 8138 & CCUG 31349	99	1e-135
13179	*Anaerococcus*	*lactolyticus*	JCM 8140 & CCUG 31351	98	1e-144
32	*Peptoniphilus*	*grossensis*	unknown	100	7e-147
268	*Peptoniphilus*	*koenoeneniae*	WAL 18898	100	7e-147
231	*Peptococcus*	*niger*	JCM 6506 & DSM 20475	100	2e-143
58	*Campylobacter*	*ureolyticus*	RIGS 9880	100	4e-143
236	*Arcanobacterium*	*pluranimalium*	M430/94/2 & 1128	96.77	2e-126
19801	*Varibaculum*	*cambriense*	DNF00696 & M380 & CCUG44998	97.51	2e-131
64	*Fusobacterium*	*nucleatum*	KCOM 2931	100	4e-143
5651	*Lactobacillus*	*iners*	DSM 13335	93	3e-136
217	*Parvimonas*	*micra*	JCM 12970 & 3119B & ATCC 33270	99	6e-108
29	*Finegoldia*	*magna*	JCM 1766	100	4e-143
6282	*Lactobacillus*	*iners*	DSM 13335	100	1e-130
118	*Schaalia*	*turicensis*	APL10	100	7e-147
40	*Prevotella*	*buccalis*	SEQ 186 & JCM 12246	100	4e-143
16	*Anaerococcus*	*tetradius*	DNF00175	100	4e-143
13	*Anaerococcus*	*obesiensis*	Ph10	100	4e-143

Supplemental Table S4. *P* value table for beta diversity PERMANOVA test for patients with EC. Samples rarefied to 515 sequencing reads. PERMANOVA test was adjusted for menopause.

BC	Lower	Uterus	Fallopian	Urine	Stool
Lower	NA	**0.027**	0.108	**0.025**	**0.001**
Uterus	**0.027**	NA	0.94	0.444	**0.001**
Fallopian	0.108	0.94	NA	0.237	**0.001**
Urine	**0.025**	0.444	0.237	NA	**0.001**
Stool	**0.001**	**0.001**	**0.001**	**0.001**	NA

Supplemental Table S5. *P* value table for beta diversity PERMANOVA test for patients without EC. Samples rarefied to 515 sequencing reads. PERMANOVA test was adjusted for menopause and pH.

BC	Lower	Uterus	Fallopian	Urine	Stool
Lower	NA	**0.02**	0.101	0.128	**0.001**
Uterus	**0.02**	NA	0.842	0.636	**0.001**
Fallopian Tube	0.101	0.842	NA	0.319	**0.001**
Urine	0.128	0.636	0.319	NA	**0.001**
Stool	**0.001**	**0.001**	**0.001**	**0.001**	NA

Supplemental Table S6. *P* value table for beta diversity correlation test for patients with EC.

BC	Lower	Uterus	Fallopian	Urine	Stool
Lower	NA	**0.01**	0.35	**0.001**	0.42
Uterus	**0.01**	NA	**0.003**	**0.001**	**0.049**
Fallopian	0.35	**0.003**	NA	0.227	0.856
Urine	**0.001**	**0.001**	0.227	NA	0.152
Stool	0.42	**0.049**	0.856	0.152	NA

Supplemental Table S7. *P* value table for beta diversity correlation test for patients without EC.

BC	Lower	Uterus	Fallopian	Urine	Stool
Lower	0	0.111	0.172	**0.001**	0.74
Uterus	0.111	0	**0.001**	0.057	0.402
Fallopian	0.172	0.001	0	**0.039**	0.639
Urine	**0.001**	0.057	**0.039**	0	**0.008**
Stool	0.74	0.402	0.639	**0.008**	0

Supplemental Table S8. Detection of *A. vaginae* by qPCR. Data are presented as number (percent). P value refers to significant presence of *A. vaginae* in cancer patients.

Atopobium vaginae	**All Patients (N=136)**	**Hyperplasia (N=3)**	**Benign (N=71)**	**Cancer (N=62)**	**P value**
Absent	111 (82)	2 (67)	58 (82)	51 (82)	0.93 (>.99)
Present	25 (18)	1 (33)	13 (18)	11 (18)	

Supplemental Table S9. Detection of *P. somerae* (OTU 28) by qPCR. Data are presented as number (percent). P value refers to significant presence of *P. somerae* in cancer patients.

Porphyromonas somerae	**All Patients (N = 146)**	**Hyperplasia (N = 8)**	**Benign (N = 73)**	**Cancer (N = 65)**	**P Value**
Absent	66 (45)	3 (37)	46 (63)	17 (26)	**$3.02E^{-7}$** (<.001)
Present	80 (55)	5 (63)	27 (37)	48 (74)	

Supplemental Table S10. Specificity, sensitivity and Area Under the Curve (AUC) values for the detection of *P. somerae* in the entire cohort and for patients at increased risk for endometrial cancer. PPV = positive predictive value, NPV = negative predictive value

	Sensitivity	Specificity	PPV	NPV	Accuracy	AUC
Overall	0.74 (0.61-0.84)	0.63 (0.51-0.74)	0.64 (0.52-0.75)	0.73 (0.6-0.83)	0.68 (0.6-0.76)	0.68 (0.61-0.76)
Obesity	0.82 (0.67-0.91)	0.44 (0.28-0.62)	0.66 (0.52-0.78)	0.65 (0.43-0.83)	0.66 (0.54-0.76)	0.63 (0.53-0.73)
Post-menopause	0.75 (0.6-0.85)	0.6 (0.36-0.8)	0.83 (0.68-0.92)	0.48 (0.28-0.68)	0.7 (0.58-0.8)	0.67 (0.55-0.8)
Obese and Post-menopause	0.83 (0.67-0.93)	0.38 (0.1-0.74)	0.86 (0.69-0.95)	0.33 (0.09-0.69)	0.75 (0.59-0.86)	0.6 (0.41-0.79)

Supplementary Table S12. Sequencing data information aggregated per patient. *Includes negative controls, samples from uterus, cervix, fallopian tubes, ovaries, peritoneal fluid, urine, vagina, and stool from patients with cancer and benign hysterectomies [$]Lower Tract = cervix and vagina swabs and scrapes [¶]These samples not used in analysis due to incomplete sampling per patient or low sequencing read count.

	All Samples*	Negative Controls (Per Sample)	Uterus	Lower Tract[$]	Stool	Urine	Peritoneal Fluid[¶]	Ovaries[¶]	Fallopian Tubes[¶]
Total Aggregated Reads	$2.5x10^7$	$9.0x10^5$	$1.0x10^6$	$2.1x10^7$	$1.9x10^6$	$8.5x10^5$	$8.6x10^4$	$1.4x10^5$	$1.4x10^5$
Maximum Reads Per Patient	$6.9x10^5$	$2.9x10^5$	$1.1x10^5$	$6.9x10^5$	$1.6x10^5$	$1.1x10^5$	$1.9x10^4$	$5.5x10^4$	$3.7x10^4$
Minimum Reads Per Patient	524	540	515	524	$1.5x10^4$	564	583	688	511
Average Reads Per Patient	$1.6x10^5$	$4.5x10^3$	$2.2x10^4$	$1.4x10^5$	$4.8x10^4$	$1.6x10^4$	$4.1x10^3$	$1.4x10^4$	$8.1x10^3$

Supplemental Table S13. Degrees of Freedom (DF) and F Values for PERMANOVA Tests.

	Pathology	Organ	Comparison	Test	DF	F Value	Pathology	Organ	Comparison	Test	DF	F Value
Alpha Diversity	Benign	Lower Tract	BMI	Shannon	1	5.816	Benign	Uterus	BMI	Shannon	1	0.551
				Observed	1	3.292				Observed	1	0.142
			pH	Shannon	1	20.569			pH	Shannon	1	2.884
				Observed	1	6.119				Observed	1	1.433
			Menopause	Shannon	1	10.079			Menopause	Shannon	1	6.426
				Observed	1	16.227				Observed	1	4.391
	Cancer, Benign	Lower Tract	Cancer vs Benign	Shannon	1	0.665	Cancer, Benign	Lower Tract	Cancer vs Benign	Shannon	1	2
				Observed	1	0.306				Observed	1	1.518
Beta Diversity	Benign	Lower Tract	BMI	UniFrac	1	1.805	Benign	Uterus	BMI	UniFrac	1	1.119
				GUniFrac	1	1.347				GUniFrac	1	0.696
				WUniFrac	1	0.937				WUniFrac	1	0.479
				BC	1	0.932				BC	1	0.899
			Menopause	UniFrac	1	2.498			Menopause	UniFrac	1	1.315
				GUniFrac	1	3.514				GUniFrac	1	1.446
				WUniFrac	1	2.13				WUniFrac	1	1.823
				BC	1	2.964				BC	1	1.134
			pH	UniFrac	1	2.037			pH	UniFrac	1	1.058
				GUniFrac	1	5.722				GUniFrac	1	1.605
				WUniFrac	1	6.795				WUniFrac	1	2.746
				BC	1	4.008				BC	1	1.508
	Cancer, Benign	Lower Tract	Cancer vs Benign	UniFrac	1	0.905	Cancer, Benign	Uterus	Cancer vs Benign	UniFrac	1	0.844
				GUniFrac	1	2.481				GUniFrac	1	1.34
				WUniFrac	1	3.801				WUniFrac	1	2.029
				BC	1	1.339				BC	1	0.905
Organ PERMANOVA Tests	Benign	Fallopian, Lower Tract, Uterus, Stool, Urine	Fallopian vs Lower	BC	1	1.808	Cancer	Fallopian, Lower Tract, Uterus, Stool, Urine	Fallopian vs Lower	BC	1	1.376
			Fallopian vs Stool	BC	1	5.2			Fallopian vs Stool	BC	1	6.359
			Fallopian vs Urine	BC	1	1.086			Fallopian vs Urine	BC	1	1.556
			Fallopian vs Uterus	BC	1	0.659			Fallopian vs Uterus	BC	1	0.565
			Lower vs Stool	BC	1	12.185			Lower vs Stool	BC	1	14.659
			Lower vs Urine	BC	1	1.582			Lower vs Urine	BC	1	1.729
			Lower vs Uterus	BC	1	2.513			Lower vs Uterus	BC	1	1.771
			Stool vs Urine	BC	1	7.886			Stool vs Urine	BC	1	11.815
			Stool vs Uterus	BC	1	7.347			Stool vs Uterus	BC	1	8.037
			Urine vs Uterus	BC	1	0.842			Urine vs Uterus	BC	1	0.973

Supplemental Table S14. Oligonucleotide sequences of primers and probes used in this study

**Specificity**	**Primer or Probe**	**Sequence (5'→3')**	**Position (bp)**	**Fluorophores**
**Eubacteria**	Forward primer	TGGAGCATGTGGTTTAATTCGA	891-912	
	Reverse primer	TGCGGGACTTAACCCAACA	1051-1033	
	Taqman probe	CACGAGCTGACGACARCCATGCA	1024-1002	6-FAM, ZEN, IOWA BLACK FQ
P. somerae	Forward primer	TGCGTAGGTGGCTGATTAAG	216-236	
	Reverse primer	AGTTTACGGCGTGGACTACC	344-325	
	Taqman Probe	CCCACGCTTTCGTGCCTCAG	301-282	6-FAM, ZEN, IOWA BLACK FQ
A. vaginae	Forward primer	AGCAGAAGCGAATCCCTAAA	1215-1235	
	Reverse primer	GTGCTGATCCGCGATTACTA	1309-1290	
	Taqman probe	CCAATCCGAACTGGGACCGG	1255-1236	NED, ZEN, IOWA BLACK FQ
